# Supplementary material for: Pragmatic multicentre randomised controlled trial evaluating the impact of a routine molecular point-of-care ‘test-and-treat’ strategy for influenza in adults hospitalised with acute respiratory illness (FluPOC): trial protocol
Source: BMJ Open. 2019 Dec 17;9(12):e031674. doi: 10.1136/bmjopen-2019-031674 (PMC6937093; doi:10.1136/bmjopen-2019-031674)
Supplement: Author's manuscript [file bmjopen-2019-031674.draft_revisions.pdf]

BMJ Open

BMJ Open is committed to open peer review. As part of this commitment we make the peer review history of every article we publish publicly available.

When an article is published we post the peer reviewers' comments and the authors' responses online. We also post the versions of the paper that were used during peer review. These are the versions that the peer review comments apply to.

The versions of the paper that follow are the versions that were submitted during the peer review process. They are not the versions of record or the final published versions. They should not be cited or distributed as the published version of this manuscript.

BMJ Open is an open access journal and the full, final, typeset and author-corrected version of record of the manuscript is available on our site with no access controls, subscription charges or pay-per-view fees (http://bmjopen.bmj.com).

If you have any questions on BMJ Open's open peer review process please email info.bmjopen@bmj.com

BMJ Open

Pragmatic multicentre randomised controlled trial evaluating the impact of a routine molecular Point-of-Care 'test-and-treat' strategy for influenza in adults hospitalised with acute respiratory illness (FluPOC): trial protocol

Journal:	*BMJ Open*
Manuscript ID	bmjopen-2019-031674
Article Type:	Protocol
Date Submitted by the Author:	15-May-2019

Complete List of Authors:	Beard, Kate; University of Southampton, Academic Unit of Clinical and Experimental Sciences, Faculty of Medicine<br>Brendish, Nathan ; University of Southampton, Academic Unit of Clinical and Experimental Sciences, Faculty of Medicine; University Hospital Southampton NHS Foundation Trust, Department of Infection<br>Malachira, Ahalya; University Hospital Southampton NHS Foundation Trust, Department of Infection<br>Mills, Samuel; University Hospital Southampton NHS Foundation Trust, Department of Infection<br>Chan, Cathleen; University Hospital Southampton NHS Foundation Trust, Department of Infection<br>Poole, Stephen; University of Southampton, Academic Unit of Clinical and Experimental Sciences, Faculty of Medicine; NIHR Southampton Biomedical Research Centre, University Hospital Southampton NHS Foundation Trust<br>Clark, Tristan; University of Southampton, Academic Unit of Clinical and Experimental Sciences, Faculty of Medicine; University Hospital Southampton NHS Foundation Trust, Department of Infection

Keywords:	Influenza, Adult, Hospitalised, Point-of-care test, Neuraminidase inhibitors

SCHOLARONE™
Manuscripts

Pragmatic multicentre randomised controlled trial evaluating the impact of a routine molecular Point-of-Care 'test-and-treat' strategy for influenza in adults hospitalised with acute respiratory illness (FluPOC): trial protocol

Kate R Beard*[1], Nathan J Brendish[1,2], Ahalya K Malachira[2], Samuel R Mills[2], Cathleen MT Chan[2], Stephen Poole[1,3], Tristan W Clark[1,2,3,4].

1. Academic Unit of Clinical and Experimental Sciences, Faculty of Medicine, University of Southampton, Southampton, UK

2. Department of Infection, University Hospital Southampton NHS Foundation Trust, Southampton, UK

3. NIHR Southampton Biomedical Research Centre, University Hospital Southampton NHS Foundation Trust, Southampton, UK

4. NIHR Post-doctoral Fellowship Programme, UK

Kate R Beard *Corresponding Author. LF100, Level F South Academic Block, Southampton General Hospital, Tremona Road, Southampton. SO16 6YD. Tel: 02381204888. Email: k.r.beard@soton.ac.uk

Ahalya K Malachira: ahalya.malachira@uhs.nhs.uk

Nathan J Brendish: nathan.brendish@uhs.nhs.uk

Samuel R Mills: samuel.mills@uhs.nhs.uk

Cathleen MT Chan: cathleen.chan@uhs.nhs.uk

Stephen Poole: stephen.poole@nhs.net

Tristan W Clark: t.w.clark@soton.ac.uk

Word count: 4802

For peer review only

Items required by the World Health Organization Trial Registration Data Set

Trial Registration: ISRCTN17197293

Date of registration: 13/11/2017

Secondary identifying numbers: RHM MED 1438, 17/SC/0368

Financial support: NIHR Post-doctoral Fellowship Programme: PDF-2016-09-061

Sponsor: University Hospital Southampton NHS Foundation Trust, (R&Doffice@uhs.nhs.uk)

Contact for public queries: Dr Tristan Clark, Associate Professor in Infectious Diseases, Clinical and Experimental Sciences, Faculty of Medicine, University of Southampton, Southampton, UK.

Tel: 02381208410. t.w.clark@soton.ac.uk

Contact for scientific queries: Dr Tristan Clark, Associate Professor in Infectious Diseases, Clinical and Experimental Sciences, Faculty of Medicine, University of Southampton, Southampton, UK.

Tel: 02381208410. t.w.clark@soton.ac.uk

Public title: Study examining the potential benefits of routine rapid accurate testing for influenza in adults with acute respiratory illness

Scientific title: Pragmatic multicentre randomised controlled trial evaluating the impact of a routine molecular Point-of-Care 'test-and-treat' strategy for influenza in adults hospitalised with acute respiratory illness (FluPOC): trial protocol

Countries of recruitment: UK

Health condition: Influenza

Intervention: Point-of-care testing with a rapid molecular test platform for influenza.

Control: Routine clinical care

For peer review only

Inclusion criteria:

- Is a patient in an Emergency Department* or Acute Medical Unit, within University Hospital Southampton NHS Foundation Trust (UHS) or Hampshire Hospitals NHS Foundation Trust (HHFT).

- Aged ≥18 years old

- Has acute respiratory illness**

- Duration of respiratory illness less than 10 days prior to hospitalisation

- Can be recruited to the study within 16 hours of presentation

*For patients in ED a decision will have already been made that the patient will be admitted.

**An episode of acute respiratory illness is defined as an acute pulmonary illness (including pneumonia, bronchitis and influenza-like illness) or an acute exacerbation of a chronic respiratory illness (including exacerbation of COPD, asthma or bronchiectasis). For the study, acute respiratory illness as a provisional, working, differential or confirmed diagnosis must be made by a treating clinician.

Exclusion criteria:

- Not fulfilling all the inclusion criteria

- A purely palliative approach being taken by the treating clinicians

- Previously included in this study and re-presenting within the last 30 days after hospital discharge

- Declines nasal / pharyngeal swabbing

- Consent declined or consultee consent declined

Study type: Multicentre, randomised controlled trial. Open label, parallel group with 1:1 allocation to intervention and control groups.

For peer review only

Randomisation: Internet based random sequence allocation using random permuted blocks (sealedenvelope.com)

Date of first enrolment: 12/12/2017

Date of last enrolment: -

Target sample size: 840

Recruitment status: ongoing

Primary Outcome: The primary outcome measure is the difference in the proportion of influenza positive patients treated appropriately with neuraminidase inhibitors (NAI) during their hospital stay (within 5 days of admission).

Key secondary outcomes*

Proportion of cases of influenza identified

Proportion of cases of all respiratory viruses detected

Proportion of all NAI use occurring in influenza positive patients

Proportion of all NAI use occurring in influenza negative patients

Time from admission to NAI commencement, hours

Duration of NAI use in influenza positive patients, days and doses

Duration of NAI use in influenza negative patients, days and doses

Proportion of patients treated with antibiotics

Proportion of patients treated with single doses or brief courses (<48 hours) of antibiotics

Duration of antibiotic use, days

For peer review only

Proportion of patients isolated in a side room

Duration of isolation facility use

Proportion of influenza cases correctly isolated

Time from admission to isolation of influenza positive cases

Time from admission to de-isolation of influenza negative cases

Duration of hospitalisation, days

Time to clinical stability, days

Time on supplementary oxygen, days

Proportion of patients with ICU or HDU admission

Duration of ICU or HDU stay, days

Proportion re-presenting to hospital within 30 days

Proportion readmitted to hospital within 30 days

In hospital, 30 and 60 day mortality

Turn-around time for viral testing

*All secondary outcome measures relate to the period of hospitalisation (up to 30 days)

For peer review only

PROTOCOL VERSION 1.1 23rd November 2017

Roles and responsibilities

Chief and Principal Investigator: Dr Tristan W Clark, Associate Professor in Infectious Diseases

Academic Unit of Clinical and Experimental Sciences, Faculty of Medicine, University of Southampton, Southampton, UK, Department of Infection, University Hospital Southampton NHS Foundation Trust, Southampton, UK, NIHR Southampton Biomedical Research Centre, University Hospital Southampton NHS Foundation Trust, Southampton, UK and NIHR Post-doctoral Fellowship Programme, UK.

Co-investigator: Dr Kate Beard, Clinical Research Fellow

Academic Unit of Clinical and Experimental Sciences, Faculty of Medicine, University of Southampton, Southampton, UK

Co-investigator: Dr Nathan J Brendish, Clinical Research Fellow

Academic Unit of Clinical and Experimental Sciences, Faculty of Medicine, University of Southampton, Southampton, UK and Department of Infection, University Hospital Southampton NHS Foundation Trust, Southampton, UK.

Co-investigator: Dr Ahalya Malachira, Clinical Research Fellow

Department of Infection, University Hospital Southampton NHS Foundation Trust, Southampton, UK.

Co-investigator: Dr Samuel Mills, Clinical Research Fellow

Department of Infection, University Hospital Southampton NHS Foundation Trust, Southampton, UK.

For peer review only

Co-investigator: Dr Cathleen Chan, Clinical Research Fellow

Department of Infection, University Hospital Southampton NHS Foundation Trust, Southampton, UK.

Co-investigator: Dr Stephen Poole, Clinical Research Fellow

Academic Unit of Clinical and Experimental Sciences, Faculty of Medicine, University of Southampton, Southampton, UK and NIHR Southampton Biomedical Research Centre, University Hospital Southampton NHS Foundation Trust, Southampton, UK

For peer review only

Abstract

Introduction

Influenza infections often remain undiagnosed in patients admitted to hospital due to lack of routine testing. When tested for, the diagnosis and treatment of influenza is often delayed due to the slow turnaround times of centralised laboratory PCR testing. Newer molecular systems, such as the FilmArray Respiratory Panel have comparable accuracy to laboratory PCR testing, and can generate a result in under 1 hour, making them potentially deployable as point-of-care tests (POCTs). High quality evidence for the impact of routine POCT for influenza on clinical outcomes is however, currently lacking. This large pragmatic multicentre randomised controlled trial aims to address this evidence gap.

Methods and Analysis

The FluPOC trial is a pragmatic, multi-centre, randomised controlled trial evaluating adults admitted to a large teaching hospital and a district general hospital with an acute respiratory illness, during influenza season. Up to 840 patients will be recruited over up to three influenza seasons, and

randomised (1:1) to receive either POCT using the FilmArray Respiratory Panel, or routine clinical care. Clinical and infection control teams will be informed of the results in real time and where influenza is detected patients will be offered neuraminidase inhibitor (NAI) treatment immediately, in accordance with national guidelines. Those allocated to standard clinical care will have a swab taken for later analysis to allow assessment of missed diagnoses. The outcomes assessment will be by retrospective case note analysis. The outcome measures include the proportion of influenza-positive patients detected and appropriately treated with NAIs, isolation facility use, antibiotic use, length of hospital stay, complications and mortality.

Ethics and Dissemination

Ethical approval was obtained from the South Central Hampshire A Ethics Committee (reference 17/SC/0368, granted 7th September 2017). Results generated from this protocol will be published in peer reviewed scientific journals and at presented at national and international conferences.

Trial Registration number

ISRCTN17197293

Key words

Influenza, Adult; Hospitalised; Point-of-care test; Neuraminidase inhibitors

Article Summary

Strengths and limitations of this study

- This is a multicentre randomised controlled trial.
- The pragmatic trial design more accurately reflects routine clinical care in a hospital setting.

For peer review only

- This is a highly relevant clinical question currently given the variable use of neuraminidase inhibitors in clinical practice, the ongoing threat of further influenza pandemics and the undefined role of influenza point-of-care testing in acute respiratory illness during influenza seasons.
- The inclusion, of some patients lacking capacity and older patients often with multiple co-morbidities, makes the trial population more generalizable to a secondary care population.
- There is ongoing uncertainty however as to how the suggested 'test-and-treat' strategy could be best implemented in clinical practice

Background

Respiratory virus burden of disease

Respiratory tract infections are the second most common cause of mortality and morbidity worldwide, [1] with viruses the most frequently detected pathogens in adults hospitalised with acute respiratory illness. [2] Seasonal influenza epidemics lead to excess hospitalisations and death due to complications including pneumonia and exacerbation of underlying cardiopulmonary conditions, and occur mainly in the elderly and patients with co-morbidity. [3-5]

Influenza: widespread but under-diagnosed

Estimates of the burden of influenza virus infection in hospitalised adults have traditionally been based on the incidence of the influenza-like-illness syndrome (ILI, defined as fever of >38°C and new respiratory symptoms) which has poor sensitivity (around 50%) and specificity (0-63%), rather than on laboratory confirmed influenza. [6-9] Patients may also present as decompensated cardiovascular disease, collapse or diabetic emergencies. [10,11] A recent Canadian study estimated that only around 1 in 14 emergency department (ED) visits due to influenza virus infection were correctly attributed to influenza. [12]

For peer review only

Sampling for respiratory viruses is generally performed on upper respiratory tract samples however several recent studies suggest that in lower respiratory tract syndromes (such as pneumonia and exacerbation of COPD) testing of upper respiratory tract samples for viruses is insensitive compared to testing lower respiratory tract samples. [13, 14]

For these reasons it is likely the burden of influenza and other respiratory viruses amongst hospitalised adults and its economic impact have been vastly under-estimated.

Neuraminidase inhibitors

Neuraminidase inhibitors (NAIs) such as oseltamivir (Tamiflu) are recommended by Public Health England (PHE) and WHO guidelines for all hospitalised adults with suspected and proven influenza infection. [15] Although there have been no randomised controlled trial evaluating the efficacy of NAIs in this group, well controlled observational data suggests that treatment of influenza with NAIs reduces mortality in hospitalised adults, especially when commenced rapidly. [16, 17] Therefore it is important that hospitalised patients with influenza are all identified and treated as soon as possible after presentation.

Previous POCTs for influenza

Rapid diagnostic tests for influenza, based on antigen detection in upper respiratory tract samples, have been available for many years but have poor diagnostic accuracy in adults, where sensitivity is around 50%. [18, 19] The poor sensitivity of these antigen-based tests has limited their clinical utility and their use was not associated with clinical or health economic benefits in a large randomised controlled trial of hospitalised adults. [20] The current gold standard diagnostic test for respiratory viruses is laboratory performed polymerase chain reaction (PCR) which is highly sensitive and specific but has a typical turnaround time for results of 24-48 hours. [21] New rapid, molecular tests have recently been developed, including the BioFire FilmArray Respiratory Panel. These molecular

For peer review only

platforms are broadly equivalent in accuracy to laboratory PCR, without the need for specialist laboratory support and expertise, can provide a result in under 1 hour and can potentially be used as a point-of-care test (POCT).

FilmArray Respiratory Panel

The FilmArray Respiratory Panel 2 (BioFire Diagnostics, Utah, USA) uses nested real-time PCR to detect around 18 respiratory viruses. The FilmArray requires only 2 minutes of "hands on" time and produces a test result in around 45 minutes. The FilmArray Respiratory Panel 2 is both FDA-cleared and CE IVD marked. The viral pathogens detected by the FilmArray Respiratory Panel 2 include: Influenza A (H1 and H3), Influenza B, Adenovirus, Coronaviruses (HKU1, NL63, 229E, OC43) Human Metapneumovirus, Human Rhinovirus/Enterovirus, Parainfluenza (types 1-4) and Respiratory Syncytial Virus. [22] The FilmArray respiratory panel is broadly equivalent in accuracy to laboratory PCR, and has been validated on nose and throat swabs, nasopharyngeal aspirates, lower respiratory tract samples, and on samples from immunocompromised patients. [23-30] These studies also show high reliability, ease of use and short turnaround times.

Clinical impact of point-of-care testing for respiratory viruses

Though viruses are the most commonly detectable pathogen in adults hospitalised with acute respiratory illness, antibiotic use is near-universal. [2] Antibiotic over-use for acute respiratory illness is partly driven by clinical uncertainty regarding the underlying aetiology; therefore early identification of viruses may prevent or shorten unnecessary antibiotic use. Previous small studies have suggested that rapid molecular viral testing might reduce antibiotic use and improve influenza antiviral use. [31, 32] We have recently shown in a large pragmatic randomised controlled trial (ResPOC) that routine molecular POCT for respiratory viruses (using the FilmArray respiratory panel) in adults presenting to hospital with acute respiratory illness is associated with a number of clinical benefits compared to routine clinical care including a reduction in unnecessary antibiotics use,

For peer review only

reduced length of hospital stay, improved influenza detection and antiviral use, and improved side room utilisation. POCT also appeared to be safe as it was not associated with an increase in adverse vents compared to routine clinical care. [33]

Alignment with global research priorities

In addition to being focused on patient and health care organisation outcomes, this clinical research is strongly aligned with several global research priority initiatives including the World Health Organisation's Battle against Respiratory Viruses (BRaVe) initiative [34] and the UK national report into antibiotic resistance. [35] The BRaVe initiative aims to catalyse multidisciplinary research on strategies to prevent and treat medically important respiratory virus infections with the goal of timely integration of research advances into public health practice. Priority areas identified include improving diagnostic tests for viral respiratory illness and improving the clinical management of patients with acute respiratory viral illness, both addressed by this study. With the growing threat of antimicrobial resistance to global public heath there is increasing need for the research and development of interventions which can help avoid unnecessary antibiotics use. [36] The data that will be generated by this trial regarding the impact of influenza POCT on antibiotic prescribing is highly aligned with this global initiative.

Added value

In addition to evaluating the clinical benefit of routine molecular POCT for influenza we will also use this opportunity to answer other important research questions including the ideal specimens to take for respiratory virus testing (upper versus lower respiratory tract specimens), the difference in viral load between these two specimen types, and whether changes in viral load over time (kinetics) in respiratory samples or the presence of viral RNA in the blood, are related to and predictive of clinical outcome.

For peer review only

Study aims and objectives

Aims

This study aims to prospectively evaluate the impact of a routine molecular Point-of-Care 'test and treat' strategy for influenza in adults hospitalised with acute respiratory illness.

Objectives

1. To evaluate the impact of routine molecular POCT on the clinical management of influenza including: the detection rate of influenza, the adherence to national (PHE) influenza guidelines, the speed and appropriateness of neuraminidase inhibitor use and the speed and appropriateness of isolation facility use.

2. To evaluate the impact of routine molecular POCT on measures of clinical effectiveness in adults hospitalised with influenza including: time to clinical stability, time on supplementary oxygen, duration of hospitalisation, adverse events and mortality.

3. To explore the differences in viral detection between upper and lower respiratory tract samples and the changes in viral load that occur over time in NAI treated and untreated patients.

Trial design

This is a pragmatic, multicentre, parallel group, open label, randomised controlled study. Groups are allocated 1:1 to the intervention arm (POCT) and control arm (routine clinical care). This protocol adheres to the SPIRIT statement. [37] The study will take place across up to three influenza seasons (as defined by national PHE surveillance programmes).

Study processes that patient-participant will undergo are summarised in Figure 1.

For peer review only

Methods

Participants, interventions and outcomes

Study setting

This is a multi-centre study based within secondary care at University Hospital Southampton NHS Foundation Trust, Southampton, UK and Royal Hampshire County Hospital, Hampshire Hospitals NHS Foundation Trust, Winchester, UK. Patients will be recruited from the Emergency Department (ED) and the Acute Medical Unit (AMU) at these hospital sites.

Eligibility criteria

Inclusion criteria

- Is a patient in an ED* or AMU, within University Hospital Southampton NHS Foundation Trust (UHS) or Hampshire Hospitals NHS Foundation Trust (HHFT)

- Aged ≥18 years old

- Has acute respiratory illness**

- Duration of respiratory illness less than 10 days prior to hospitalisation

- Can be recruited to the study within 16 hours of presentation

*For patients in ED a decision will have already been made that the patient will be admitted.

**An episode of acute respiratory illness is defined as an acute pulmonary illness (including pneumonia, bronchitis and influenza-like illness) or an acute exacerbation of a chronic respiratory illness (including exacerbation of COPD, asthma or bronchiectasis). For the study, acute respiratory

For peer review only

illness as a provisional, working, differential or confirmed diagnosis must be made by a treating clinician.

Exclusion criteria

- Not fulfilling all the inclusion criteria

- A purely palliative approach being taken by the treating clinicians

- Previously included in this study and re-presenting within the last 30 days after hospital discharge

- Declines nasal / pharyngeal swabbing

- Consent declined or consultee consent declined

Concurrent, prior or subsequent enrolment in an observational study is not necessarily an exclusion criterion; this is at the discretion of the chief investigator and will be assessed on a case-by-case basis.

Interventions

For both control and intervention groups:

A respiratory sample will be collected by a member of research staff. A combined nose and throat swab will be taken and placed into viral transport medium. If feasible, a lower respiratory tract sample (sputum) will also be obtained.

Clinical and demographic data will be collected at the time of enrolment. Outcome data will be collected from case notes and electric health records retrospectively.

A blood sample (maximum of 21ml) will be obtained from all willing participants, processed in the NIHR Southampton Clinical Research Facility lab and stored at -80°C pending further analysis. All respiratory samples left-over from FilmArray testing may be frozen and stored for further study.

For peer review only

Participant consent for this is included in the consent form. Patients may be approached for additional samples to be collected and stored for further study (see below for schedule of additional samples).

A letter detailing that the patient has been included in this trial will be sent to the patient's general practitioner for information only.

For those randomised to the interventional arm:

The nose and throat swab in viral transport medium will be analysed promptly on the FilmArray Respiratory Panel as per training delivered by the apparatus manufacturer, used as a point-of-care test. FilmArray machines will be located in the clinical areas for this purpose. Test results are generated in about 1 hour. Where lower respiratory tract samples are also obtained these will also be tested on the FilmArray in parallel with the nose and throat swab or sequentially.

The results of the test will be documented in the patient's case notes and a member of the clinical team responsible for the patient will be directly informed. The participant or consultee will also be informed of the result where appropriate. The infection prevention and control team will be informed of the result within 4 hours of testing. In the event of influenza A or B being detected the participant will be offered treatment with NAIs as per national guidelines. It is known that the sensitivity of virus detection may be greater with lower respiratory tract samples versus upper tract samples in certain patients. Therefore in patients where lower respiratory tract samples are obtained in addition to the upper respiratory tract samples, if either of the swabs are positive they will treated as infected and be reported to the clinical team and infection control team as above.

For those randomised to the control group

These patients will be managed according to standard clinical care as directed by their treating clinicians. Laboratory respiratory virus testing will be at the discretion of the responsible clinical team and where performed will be using laboratory PCR (in the onsite laboratory). The respiratory

For peer review only

samples collected at enrolment will be stored at -80°C and subsequently tested by the FilmArray respiratory panel at least 30 days after collection. This will allow direct comparison of pathogens between the groups (i.e. an estimate of missed diagnoses in the routine clinical care group) but will not influence participant care.

Additional procedures

For participants randomised to receive the intervention, who subsequently have a pathogen detected on the FilmArray respiratory panel and for all participants randomised to routine clinical care, additional respiratory samples +/- an additional blood test (maximum 10mls) may be specifically requested from the participants to study temporal changes in viral load, according to the schedule below.

Schedule

The nose and throat swab and lower respiratory tract samples listed here are in addition to samples taken at enrolment (day 1) and are collected at five further time points: day 2 to 3, day 4 to 5, day 6 to 7, day 8 to 10 and day 11-14+. An additional blood sample (maximum of 10mls) may be taken at day 5 to 7. The participant may decline all or any of these additional tests.

Outcomes

Primary outcome

The primary outcome measure is the difference in the proportion of influenza positive patients treated appropriately with neuraminidase inhibitors (NAI) during their hospital stay (within 5 days of admission).

Secondary Outcomes

Proportion of cases of influenza identified

For peer review only

Proportion of cases of all respiratory viruses detected

Proportion of all NAI use occurring in influenza positive patients

Proportion of all NAI use occurring in influenza negative patients

Median time from admission to NAI commencement, hours

Median duration of NAI use in influenza positive patients, days and doses

Median duration of NAI use in influenza negative patients, days and doses

Proportion of patients treated with antibiotics

Proportion of patients treated with single doses or brief courses (<48 hours) of antibiotics

Median duration of antibiotic use, days

Proportion of patients isolated in a side room

Median duration of isolation facility use

Proportion of influenza cases correctly isolated

Median time from admission to isolation of influenza positive cases

Median time from admission to de-isolation of influenza negative cases

Median duration of hospitalisation, days

Median time to clinical stability*, days

Median time on supplementary oxygen, days

Proportion of patients with ICU or HDU admission

Median duration of ICU or HDU stay, days

Proportion re-presenting to hospital within 30 days

Proportion readmitted to hospital within 30 days

Proportion of in-hospital, 30 and 60 day mortality

Median turn-around time for viral testing

For peer review only

All outcomes are measured for the duration of hospitalisation or up to 30 days (whichever is shortest) unless specified otherwise and include medication (antibiotics and NAIs) that patients are discharged home with.

*Defined as: temperature ≤37.8C, respiratory rate ≤24 breaths per minute, heart rate ≤100 beats per minute, oxygen saturation ≥90% without the use of supplementary oxygen, systolic blood pressure ≥90mmHg and normal mental status for at least 24 hours. [38]

Exploratory Outcomes

Difference in influenza detection between upper and lower respiratory tract samples

Difference in influenza viral load between upper and lower respiratory tract samples

Changes in influenza viral load over time (kinetics) in respiratory tract samples and blood

Response rate on the ordinal hospital recovery scale (HRS) at day 5 and 7

Participant Timeline

Participants are identified in the AMU and ED according to eligibility criteria by research staff. Following gaining written consent participants are immediately randomised to the intervention group or the control group. For those patients randomised to the intervention group a nose/throat swab is collected by a study doctor or research nurse and this is tested on the FilmArray machine. If the patient is able to produce a sputum sample, this sample will also be tested on the FilmArray machine. Results are generated in approximately 45 minutes and are immediately communicated to the clinical team. Clinical data is collected prospectively and retrospectively for both groups. Further nose/throat swabs, sputum samples and blood tests may be collected from patients who remain inpatients on serial follow up visits up to day 14.

For peer review only

Sample size

Proposed sample size

The study will recruit up to 840 patient-participants: about 280 per season for three consecutive influenza seasons. With 1:1 allocation to groups, there will be 420 patients per group. On the conservative assumption of a 25% positivity rate for influenza during influenza season (i.e. ~100 influenza positive patients in each group) the group sizes would give a 90% power at a 0.05 significance level to detect a difference of 20% in appropriate neuraminidase inhibitor use (from 65% to 85%) and an 80% power to detect a difference of 17%. In our previous work 91% of influenza positive patients randomised to POCT were appropriately treated with NAI versus 65% with routine clinical care, a difference of 26% (95%CI 10 to 43). We suspect that the difference may be larger in this study as there will likely be a number of undiagnosed (and therefore untreated) cases in the control group revealed by testing them with the FilmArray at a later date. In the event of higher numbers of influenza positive patients being recruited in the first two seasons, the study may be stopped prematurely (once 100 influenza positive patients have been recruited to the intervention group).

Recruitment and Screening

Screening

Eligible patients in the acute medical unit (AMU) and the emergency department (ED) will be identified by research staff via regular review of the comprehensive admissions IT systems daily. Recruitment will run for up to 3 years to include 3 seasonal influenza seasons between December 2017 and May 2020.

For peer review only

Consent

The study team (research nurse, research fellow or the principal investigator) will obtain informed consent for those fulfilling eligibility criteria and willing to be recruited for those with capacity, or assent via consultee for those without capacity, as per the dedicated study forms. In view of the acute nature of patients' illnesses, the potential benefits of rapid identification of viruses including prompt neuraminidase inhibitor use for influenza, the usual 24-hour consideration period for a participant or consultee will not apply.

Discussion of the study will be provided to patients, or their consultee for those lacking capacity, by study staff. This includes supply of a participant information sheet for the participant or witness to read and retain. Consent is also acquired to obtain and store specimens (nose/throat swab, sputum sample, blood tests) for future research studies.

Each patient will be assumed to have capacity unless it is established that they lack capacity. For patients unable to consent for themselves, this study complies with the Mental Capacity Act 2005 and in such cases, the patient's family member, carer or friend may be asked to act as the personal consultee and provide assent. In the event of a personal consultee not being available a nominated consultee (usually the consultant caring for the patient and independent from the study) will be asked if they would provide assent. Both the person taking assent and the consultee must personally sign and date the relevant form.

Assignment of interventions

Sequence generation, allocation concealment and implementation

Following screening and obtaining informed written consent from an eligible patient, a unique identification number will be assigned consecutively. A research team member will then use this identification number to randomise the participant to either the intervention or control group, via

For peer review only

an internet-based randomisation service (sealedenvelope.com). This service uses permuted blocks of varying sizes and generates a randomisation code which corresponds to one of the study arms. Research staff will implement this allocation sequence, and use the allocation code generated from sealedenvelope.com to assign the patients.

Blinding

As this is a pragmatic trial of a diagnostic strategy where the results of the test must be communicated to clinical and infection control teams no attempt at blinding trial participants, research staff or care providers will be made. Data analysts will be blinded to group allocation.

For peer review only

Data collection, management and analysis

Data collection methods

Demographic and clinical data will be collected for all patients at enrolment from paper and electronic medical records including: age, sex, ethnicity, smoking status, vaccination status, co-morbidities, medication use, symptoms, duration of illness prior to hospitalisation, observations (pulse rate, respiratory rate, blood pressure, oxygenation status), laboratory results, radiology results, antimicrobial and antiviral use prior to hospitalisation and provisional diagnosis. Data will be recorded on a standardised Case Report Form (CRF) and transferred to a secure electronic database. Once patients have been discharged or after 30 days (whichever is soonest), outcome data will be collected retrospectively from electronic and physical case notes, electronic prescribing systems and laboratory and radiological results systems including: use of antivirals, duration of antivirals, time from assessment to antiviral use, use of antibiotics, duration of antibiotic use, use of side room facilities, time from assessment to isolation facility use, time to clinical stability, duration of supplementary oxygen use, duration of hospitalisation, complications including ICU and HDU

admission, representation and readmission to hospital within 30 days, final diagnosis and mortality. The number of diagnostic tests and procedures performed will also be recorded and data will also be collected on the turnaround time of respiratory virus test results in each group. Patients withdrawn from the study will have no further data collected.

Data Management

The study will be conducted in compliance with the approved protocol, relevant International Conference on Harmonisation (ICH) and Good Clinical Practice (GCP) regulations and standard operating procedures. Data will be evaluated for protocol compliance and accuracy in reference to the source documents.

The subjects' anonymity will be maintained. The study team will keep a log of each subject's name, hospital ID number, date of birth, and unique participant trial number. The participant details will be recorded on the secure NHS Edge system in a similar manner, including NHS number. This participant trial number is used on documents after screening to maintain confidentiality. Documents that are not anonymous (e.g. signed informed consent forms) will be maintained separately, in strict confidence and in accordance with the Data Protection Act 1998. A secure database will be used to enter data from the CRFs at the completion of the study, followed by data lock.

The study staff will be responsible for entering study data in the CRF. It is the investigators' responsibility to ensure the accuracy of the data entered in the CRF.

Only the research study team will know the identity of subjects and have access to the list linking participant details to the participant trial number.

For peer review only

Statistical methods

Analysis will be by intention-to-treat. The framework is superiority. Trial results will be reported in accordance with the CONSORT statement. [39]

Statistical analysis will be performed by a dedicated medical statistician from the University of Southampton, independent from the study team. No interim analysis is currently planned. Patients tested with the rapid molecular diagnostic test will be compared with patients managed according to standard clinical care using standard descriptive and comparative statistical methods using Prism version 6.0 (GraphPad Software Inc; La Jolla, California), and Stata version 13·1 (StataCorp, College Station, Texas).

Missing data was minimal (<2%) in the CI's previous POCT study involving 720 patients presenting to secondary care, and therefore is not expected to be significant issue in this trial. The use of multiple imputation will be considered should missing data exceed 10% for the primary outcome.

Summaries of all baseline characteristics will be presented using means and standard deviations, medians and interquartile ranges, or frequencies and percentages, as appropriate. The intervention and control groups will be compared using chi-square tests for equality of proportions for binary data (e.g. proportion of cases of influenza detected) and using independent-samples t-tests or non-parametric equivalent as appropriate for continuous data (e.g. duration of antibiotics, test turn-around time etc).

Primary outcome

For the primary outcome, difference in proportions and unadjusted odds ratios will be used to compare the groups. The effect of group on the primary outcome will be further assessed using multiple logistic regression to control for the following covariates: age, sex, influenza vaccine status, receipt of antibiotics prior to admission, duration of illness, comorbidity, temperature, CRP, severity of illness, turnaround time and clinical group. For secondary outcomes the intervention and control

For peer review only

groups will be compared using chi-square tests for equality of proportions for binary data (including proportions) and using t-tests and non-parametric equivalent tests for continuous data (e.g. turnaround time) as appropriate. The effect of group on certain secondary outcomes will be further assessed using multiple regression analysis controlling for the same covariates as listed above for the primary outcome.

In view of the clinical heterogeneity of influenza positive patients, heterogeneity of effect among different clinical subgroups is anticipated *a priori* and therefore a pre-planned subgroup analysis for the certain key secondary outcome measures will be undertaken based on age, clinical group (exacerbation of asthma, exacerbation of COPD, pneumonia, etc) and NAI treatment. The interaction between clinical subgroups and allocation group will be assessed in the multiple regression models detailed above.

Monitoring

Data monitoring

This trial was reviewed by the sponsor. On the basis of the very low risk of harms associated with the intervention in this non-CTIMP trial, no data monitoring committee or interim analysis is planned. A trial management committee will meet regularly and oversee the trial.

Harms

The risks of respiratory tract sampling and additional blood tests being taken are minimal and where occurring are likely to be mild. No additional adverse events related to POCT for respiratory viruses are anticipated. However active monitoring and reporting of severe adverse events will be undertaken. A Serious Adverse Event (SAE) is any adverse event that:

For peer review only

- Results in death

- Is life-threatening

- Requires hospitalisation or prolongation of existing hospitalisation

- Results in persistent or significant disability or incapacity

- Consists of a congenital anomaly or birth defect

Participants already admitted to AMU or are in ED but with a decision already made to admit, are considered already hospitalised. However, an adverse event leading to prolongation of their existing hospitalisation will be counted as an SAE.

Auditing

Regular monitoring by the sponsor will be performed according to ICH GCP. Data will be evaluated with regards to accuracy in relation to source documents and compliance with the protocol. Following written standard operating procedures, the monitors will verify that the clinical trial is conducted and data are generated, documented and reported in a manner that is compliant with the protocol, GCP and applicable regulatory requirements.

Protocol amendments

There has been one amendment made to the protocol. This was the addition of Hampshire Hospitals NHS Foundation Trust as a potential site and removal of mention of 'single centre.' The current protocol version is 1.1, dated 23rd November 2017. This amendment has been communicated to the investigators and the to trial registries. The local study reference is RHM MED 1438.

For peer review only

Confidentiality

All data will be anonymised to ensure patient confidentiality is protected. A unique study number will be used to identify participant data in the CRFs and database. Serious Adverse Events will be reported in line with GCP and regulatory requirements. All study staff have been trained in GCP. Data will be kept securely and only the investigators and sponsor's representative (monitor) have access to the data.

Access to data

Full access to the data set will be held by the principal investigator, co-investigators and independent statistician only. The data set may be made available to other parties on request.

Dissemination policy

Authorship of this manuscript and those resulting from this protocol will follow the ICMJE recommendations and CONSORT statement where appropriate, and there are no plans to use professional writers. There is no intention to make the data set publically available. Outside of the study team and regulators, the full protocol will only be made available at the discretion of the chief investigator. Multiple publications are expected to result from the data and samples collected, and these publications must acknowledge this trial and the study team as appropriate.

Patient and public involvement

Discussion with members of the public and the results of questionnaire from hospitalised patients helped inform the design of this trial.

The emphasis on informing the patients, in addition to the clinical teams, of the POCT result is a direct result of our engagement with patients. Patients and the public will also be involved in the reporting of this research.

For peer review only

Discussion

We hypothesise that the use of a routine molecular POC 'test and treat' strategy for influenza in adults hospitalised with acute respiratory illness will improve adherence to national guidelines for the management of influenza and may improve patient outcomes. We expect increased, timely detection of influenza and that this will lead to improved use of NAI treatment. Rapid and appropriate use of NAI treatment in influenza may also lead to decreased complication and mortality and may shorten length of stay in hospital. The rapid detection of viruses may improve isolation facility use within the NHS with subsequent reduction in nosocomial transmission of viruses and improvements in patient flow through acute areas. The use of a POCT for respiratory viruses may also identify patients where unnecessary antibiotic use can be safely discontinued, promoting antibiotic stewardship and reducing antimicrobial resistance.

Declarations

Ethics approval

Prior to commencing the study, approval was obtained from the South Central Hampshire A Ethics Committee (reference 17/SC/0368). A protocol amendment has been approved by the ethics committee for the addition of Hampshire Hospitals NHS Foundation Trust as a potential site and the removal of mention of 'single centre' (current protocol version 1.1, date 23rd November 2017). The local study reference number is RHM MED 1438.

Consent for publication

Not applicable

For peer review only - http://bmjopen.bmj.com/site/about/guidelines.xhtml

Availability of data and material

Not applicable

Competing interests

The principal investigator and co-investigators declare they have no competing interests relating to this study.

Funding

The study is funded by NIHR Post-doctoral Fellowship Programme, grant number: PDF-2016-09-061. The testing units and equipment for this study will be leased or purchased independently from a UK distributor using funds provided by NIHR Post Doctoral Fellowship Programme (PDF-2016-09-061). NIHR Clinical Research Network, Wessex provided salary contributions for clinical fellows to support this trial. The study is sponsored by University Hospital Southampton NHS Foundation Trust.

The study sponsor, study funders and manufacturers of the FilmArray platform had no involvement in the conception, design or running of this study, and will have no involvement in the analysis of the data or writing of subsequent manuscripts for publication.

The views expressed in this publication are those of the author(s) and not necessarily those of the NHS, the National Institute for Health Research or the Department of Health.

Author Contributions

TWC conceived the study and is the chief and principal investigator. TWC leads conduct of trial, and protocol manuscript drafting. TWC and NJB designed the study. TWC and NJB wrote the protocol. KRB drafted the protocol manuscript, and contributes to overseeing conduct of trial and patient recruitment. NJB, AKM, SRM, CMTC and SP contributed to protocol manuscript drafting and patient recruitment. All authors read and approved the final manuscript.

For peer review only

Acknowledgments

We would like to thank patients and staff in the Emergency Department and Acute Medical Unit and other inpatient wards at University Hospital Southampton NHS Foundation Trust and Hampshire Hospitals NHS Foundation Trust for making the study possible.

Abbreviations

AMU: Acute medical unit

CI: Chief Investigator

CRF: Case Report Form

ED: Emergency department

GCP: Good Clinical Practice

HHFT: Hampshire Hospitals NHS Foundation Trust

ICH: International Conference on Harmonisation

ICU: Intensive care unit

HDU: High dependency unit

NAI: Neuraminidase inhibitor

PHE: Public Health England

PI: Principal Investigator

POCT: Point-of-Care Test

For peer review only

SAE: Serious Adverse Event

UHS: University Hospital Southampton NHS Foundation Trust

Figure 1

Participant flow through the study

References

1. Lozano R, Naghavi M, Foreman K, et al. Global and regional mortality from 235 causes of death for 20 age groups in 1990 and 2010: a systematic analysis for the Global Burden of Disease Study 2010. *Lancet*2012;380:2095–128.

2. Clark TW, Medina MJ, Batham S, et al. Adults hospitalised with acute respiratory illness rarely have detectable bacteria in the absence of COPD or pneumonia; viral infection predominates in a large prospective UK sample. *J. Infect* 2014;69:507–15.

3. Thompson WW, Shay DK, Weintraub E, et al. Mortality associated with influenza and respiratory syncytial virus in the United States. *JAMA*2003;289:179–86.

4. Thompson WW, Shay DK, Weintraub E, et al. Influenza-associated hospitalizations in the United States. *JAMA*2004;292:1333–40.

5 Irwin DE, Weatherby LB, Huang WY, et al. Impact of patient characteristics on the risk of influenza/ILI-related complications. *BMC Health Serv Res*2001;1:8.

6. Dool C van den, Hak E, Wallinga J, et al. Symptoms of influenza virus infection in hospitalized patients. *Infect Control Hosp Epidemiol*2008;29:314–9.

For peer review only

7. Babcock HM, Merz LR, Dubberke ER, et al. Case-control study of clinical features of influenza in hospitalized patients. *Infect Control Hosp Epidemiol*2008;29:921–6.

8. Babcock HM, Merz LR, Fraser VJ. Is influenza an influenza-like illness? Clinical presentation of influenza in hospitalized patients. *Infect Control Hosp Epidemiol*2006;27:266–70.

9. Call SA, Vollenweider MA, Hornung CA, et al. Does this patient have influenza? *JAMA* 2005;293:987–97.

10. Falsey AR, Hennessey PA, Formica MA, et al. Respiratory syncytial virus infection in elderly and high-risk adults. *N Engl J Med*2005;352:1749–59.

11. Warren-Gash C, Smeeth L, Hayward AC. Influenza as a trigger for acute myocardial infarction or death from cardiovascular disease: a systematic review. Lancet Infect Dis. 2009;9:601–10.

12. Schanzer DL, Schwartz B. Impact of seasonal and pandemic influenza on emergency department visits, 2003-2010, Ontario, Canada. *Acad Emerg Med*2013;20:388–97.

13. López Roa P, Rodríguez-Sánchez B, Catalán P, et al. Diagnosis of influenza in intensive care units: lower respiratory tract samples are better than nose-throat swabs. *Am J Respir Crit Care Med*2012;186:929-30.

14. Falsey AR, Formica MA, Walsh EE. Yield of sputum for viral detection by reverse transcriptase PCR in adults hospitalized with respiratory illness. *J Clin Microbiol* 2012;50:21-4.

15. PHE guidance on use of antiviral agents for the treatment and prophylaxis of seasonal influenza. Public Health England (2015-16). Version 6.0. September 2015.

16. Muthuri SG, Venkatesan S, Myles PR, et al. Effectiveness of neuraminidase inhibitors in reducing mortality in patients admitted to hospital with influenza A H1N1pdm09 virus infection: a meta-analysis of individual participant data. *Lancet Respir Med*2014;2:395–404.

For peer review only

17. Katzen j, Kohn R, Houk JL et al. Early Oseltamivir After Hospital Admission Is Associated With Shortened Hospitalization: A 5-Year Analysis of Oseltamivir Timing and Clinical Outcomes. *Clin Infect Dis*2018 Published Online First 9 October 2018. doi: 10.1093/cid/ciy860.

18. Evaluation of rapid influenza diagnostic tests for detection of novel influenza A (H1N1) Virus - United States, 2009. *MMWR Morb Mortal Wkly Rep*2009;58:826–9.

19. Chartrand C, Leeflang MM, Minion J, et al. Accuracy of rapid influenza diagnostic tests: a meta-analysis. *Ann Intern Med*2012;156:500–11.

20. Nicholson KG, Abrams KR, Batham S, et al. Randomised controlled trial and health economic evaluation of the impact of diagnostic testing for influenza, respiratory syncytial virus and Streptococcus pneumoniae infection on the management of acute admissions in the elderly and high-risk 18- to 64-year-olds. *Health Technol Assess* 2014;18:1e274.

21. Mackay IM. Real-time PCR in the microbiology laboratory. *Clin Microbiol Infect* 2004;10:190–212.

22. BioFire Diagnostics. FilmArray® Respiratory Panel Information Sheet.

23. Popowitch EB, O'Neill SS, Miller MB. Comparison of the Biofire FilmArray RP, Genmark eSensor RVP, Luminex xTAG RVPv1, and Luminex xTAG RVP fast multiplex assays for detection of respiratory viruses. *J Clin Microbiol*2013;51:1528–33.

24. Poritz MA, Blaschke AJ, Byington CL, et al. FilmArray, an automated nested multiplex PCR system for multi-pathogen detection: development and application to respiratory tract infection. *PLoS ONE*2011;6:e26047.

25. Butt SA, Maceira VP, McCallen ME, et al. Comparison of three commercial RT-PCR systems for the detection of respiratory viruses. *J Clin Virol*2014;61:406–10.

26. Pierce VM, Elkan M, Leet M, et al. Comparison of the Idaho Technology FilmArray system to real-time PCR for detection of respiratory pathogens in children. *J Clin Microbiol*2012;50:364–71.

For peer review only

27. Loeffelholz MJ, Pong DL, Pyles RB, et al. Comparison of the FilmArray Respiratory Panel and Prodesse Real-Time PCR Assays for Detection of Respiratory Pathogens. *J Clin Microbiol*2011;49:4083-8.

28. Ruggiero P, McMillen T, Tang YW, et al. Evaluation of the BioFire FilmArray Respiratory Panel and the GenMark eSensor Respiratory Viral Panel on Lower Respiratory Tract Specimens. *J Clin Microbiol* 2014;52:288-90.

29. Hammond SP, Gagne LS, Stock SR, et al. Respiratory virus detection in immunocompromised patients with FilmArray respiratory panel compared to conventional methods. *J Clin Microbiol*2012;50:3216–21.

30. Doern CD, Lacey D, Huang R, et al. Evaluation and Implementation of FilmArray Version 1.7 for Improved Detection of Adenovirus Respiratory Tract Infection. *J Clin Microbiol* 2013;51:4036-9.

31. Branche AR, Walsh EE, Vargas R, et al. Serum Procalcitonin Measurement and Viral Testing to Guide Antibiotic Use for Respiratory Infections in Hospitalized Adults: A Randomized Controlled Trial. *J Infect Dis*2015;212:1692-700.

32. Dugas AF, Valsamakis A, Atreya MR, et al. Clinical diagnosis of influenza in the ED. *Am J Emerg Med*2015;33:770-5.

33. Brendish NJ, Malachira AK, Armstrong L, et al. Routine molecular point-of-care testing for respiratory viruses in adults presenting to hospital with acute respiratory illness (ResPOC): a pragmatic, open-label, randomised controlled trial. *Lancet Respir Med* 2017;5:401-411.

34. Legand A, Hayden FG. Addressing the public health burden of respiratory viruses: the Battle against Respiratory Viruses (BRaVe) Initiative. Future Virology. 2013;8:953–968.

35. Jim O'Neil (chair). Tackling drug-resistant infections globally: Final report and recommendations. 2016.

For peer review only

36. WHO. Fact sheet: antimicrobial resistance. https://www.who.int/en/news-room/fact-sheets/detail/antimicrobial-resistance (accessed 4 May 2019)

37. Chan A-W, Tetzlaff JM, Altman DG, et al. SPIRIT 2013 Statement: Defining standard protocol items for clinical trials. *Ann Intern Med*2013;158:200-207.

38. Halm EA, Fine MJ, Marrie TJ, et al. Time to clinical stability in patients hospitalized with community-acquired pneumonia: implications for practice guidelines. *JAMA*1998;279:1452–1457.

39. Schulz KF, Altman DG, Moher D, for the CONSORT Group. CONSORT 2010 Statement: updated guidelines for reporting parallel group randomised trials. *BMC Med*2010;8:18.

For peer review only

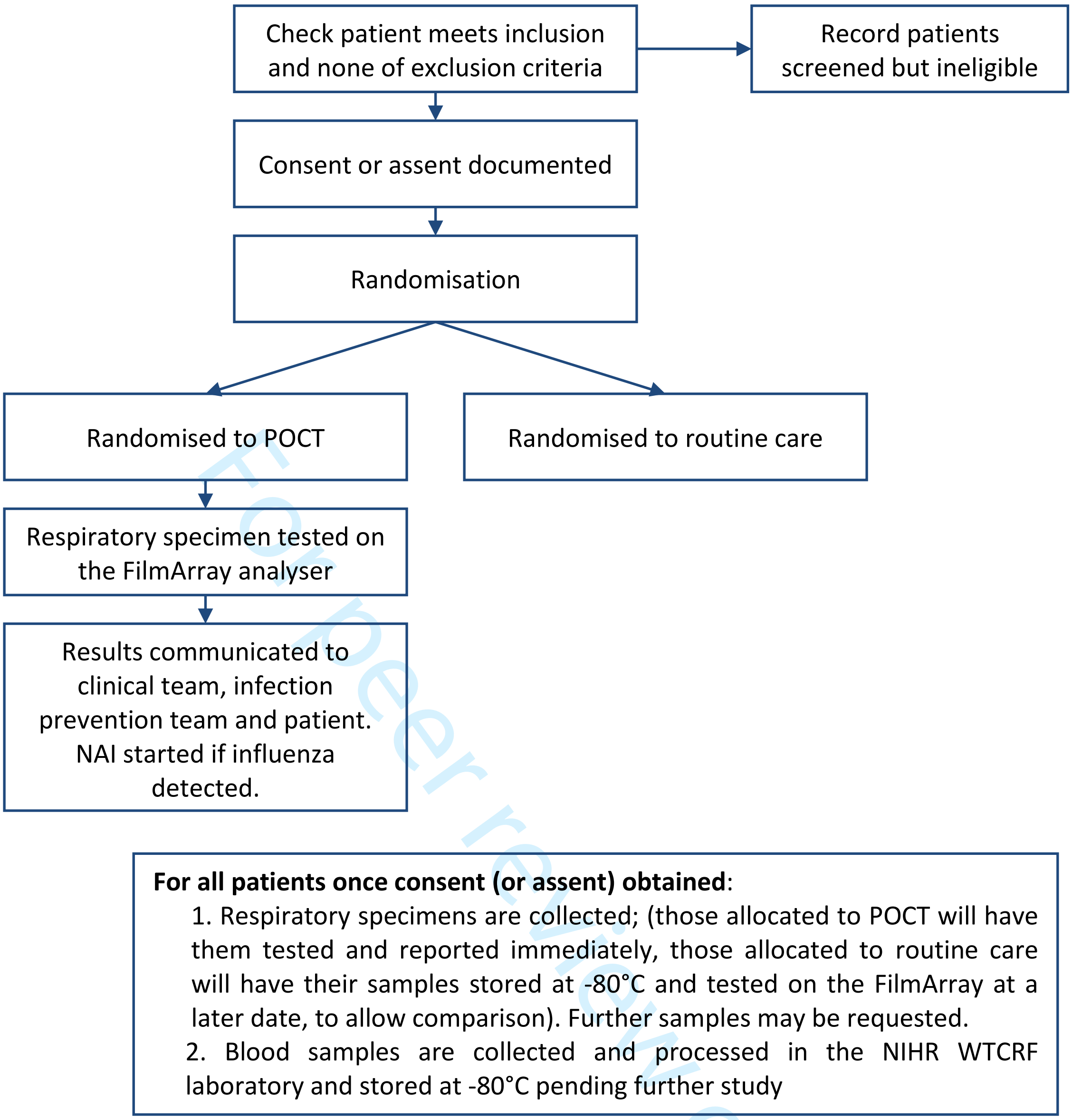

For peer review only

STANDARD PROTOCOL ITEMS: RECOMMENDATIONS FOR INTERVENTIONAL TRIALS

SPIRIT 2013 Checklist: Recommended items to address in a clinical trial protocol and related documents*

Section/item	Item No	Description	Addressed on page number
**Administrative information**			
Title	1	Descriptive title identifying the study design, population, interventions, and, if applicable, trial acronym	1, 2
Trial registration	2a	Trial identifier and registry name. If not yet registered, name of intended registry	2, 8
	2b	All items from the World Health Organization Trial Registration Data Set	2-5
Protocol version	3	Date and version identifier	6, 26
Funding	4	Sources and types of financial, material, and other support	2, 29
Roles and responsibilities	5a	Names, affiliations, and roles of protocol contributors	1, 6-7, 29
	5b	Name and contact information for the trial sponsor	2, 29
	5c	Role of study sponsor and funders, if any, in study design; collection, management, analysis, and interpretation of data; writing of the report; and the decision to submit the report for publication, including whether they will have ultimate authority over any of these activities	29
	5d	Composition, roles, and responsibilities of the coordinating centre, steering committee, endpoint adjudication committee, data management team, and other individuals or groups overseeing the trial, if applicable (see Item 21a for data monitoring committee)	Not applicable

For peer review only

Introduction

Background and rationale	6a	Description of research question and justification for undertaking the trial, including summary of relevant studies (published and unpublished) examining benefits and harms for each intervention	9-12
	6b	Explanation for choice of comparators	9-12
Objectives	7	Specific objectives or hypotheses	13
Trial design	8	Description of trial design including type of trial (eg, parallel group, crossover, factorial, single group), allocation ratio, and framework (eg, superiority, equivalence, noninferiority, exploratory)	13

Methods: Participants, interventions, and outcomes

Study setting	9	Description of study settings (eg, community clinic, academic hospital) and list of countries where data will be collected. Reference to where list of study sites can be obtained	14
Eligibility criteria	10	Inclusion and exclusion criteria for participants. If applicable, eligibility criteria for study centres and individuals who will perform the interventions (eg, surgeons, psychotherapists)	14-15
Interventions	11a	Interventions for each group with sufficient detail to allow replication, including how and when they will be administered	15-17
	11b	Criteria for discontinuing or modifying allocated interventions for a given trial participant (eg, drug dose change in response to harms, participant request, or improving/worsening disease)	Not applicable
	11c	Strategies to improve adherence to intervention protocols, and any procedures for monitoring adherence (eg, drug tablet return, laboratory tests)	Not applicable
	11d	Relevant concomitant care and interventions that are permitted or prohibited during the trial	16-17
Outcomes	12	Primary, secondary, and other outcomes, including the specific measurement variable (eg, systolic blood pressure), analysis metric (eg, change from baseline, final value, time to event), method of aggregation (eg, median, proportion), and time point for each outcome. Explanation of the clinical relevance of chosen efficacy and harm outcomes is strongly recommended	17-19
Participant timeline	13	Time schedule of enrolment, interventions (including any run-ins and washouts), assessments, and visits for participants. A schematic diagram is highly recommended (see Figure)	19

For peer review only - http://bmjopen.bmj.com/site/about/guidelines.xhtml

Sample size	14	Estimated number of participants needed to achieve study objectives and how it was determined, including clinical and statistical assumptions supporting any sample size calculations	20
Recruitment	15	Strategies for achieving adequate participant enrolment to reach target sample size	20

Methods: Assignment of interventions (for controlled trials)

Allocation:			
Sequence generation	16a	Method of generating the allocation sequence (eg, computer-generated random numbers), and list of any factors for stratification. To reduce predictability of a random sequence, details of any planned restriction (eg, blocking) should be provided in a separate document that is unavailable to those who enrol participants or assign interventions	21-22
Allocation concealment mechanism	16b	Mechanism of implementing the allocation sequence (eg, central telephone; sequentially numbered, opaque, sealed envelopes), describing any steps to conceal the sequence until interventions are assigned	21-22
Implementation	16c	Who will generate the allocation sequence, who will enrol participants, and who will assign participants to interventions	21-22
Blinding (masking)	17a	Who will be blinded after assignment to interventions (eg, trial participants, care providers, outcome assessors, data analysts), and how	22
	17b	If blinded, circumstances under which unblinding is permissible, and procedure for revealing a participant's allocated intervention during the trial	Not applicable

Methods: Data collection, management, and analysis

Data collection methods	18a	Plans for assessment and collection of outcome, baseline, and other trial data, including any related processes to promote data quality (eg, duplicate measurements, training of assessors) and a description of study instruments (eg, questionnaires, laboratory tests) along with their reliability and validity, if known. Reference to where data collection forms can be found, if not in the protocol	22-23
	18b	Plans to promote participant retention and complete follow-up, including list of any outcome data to be collected for participants who discontinue or deviate from intervention protocols	22-23

For peer review only - http://bmjopen.bmj.com/site/about/guidelines.xhtml

Data management	19	Plans for data entry, coding, security, and storage, including any related processes to promote data quality (eg, double data entry; range checks for data values). Reference to where details of data management procedures can be found, if not in the protocol	23
Statistical methods	20a	Statistical methods for analysing primary and secondary outcomes. Reference to where other details of the statistical analysis plan can be found, if not in the protocol	24-25
	20b	Methods for any additional analyses (eg, subgroup and adjusted analyses)	24-25
	20c	Definition of analysis population relating to protocol non-adherence (eg, as randomised analysis), and any statistical methods to handle missing data (eg, multiple imputation)	24
**Methods: Monitoring**			
Data monitoring	21a	Composition of data monitoring committee (DMC); summary of its role and reporting structure; statement of whether it is independent from the sponsor and competing interests; and reference to where further details about its charter can be found, if not in the protocol. Alternatively, an explanation of why a DMC is not needed	25
	21b	Description of any interim analyses and stopping guidelines, including who will have access to these interim results and make the final decision to terminate the trial	25
Harms	22	Plans for collecting, assessing, reporting, and managing solicited and spontaneously reported adverse events and other unintended effects of trial interventions or trial conduct	25-26
Auditing	23	Frequency and procedures for auditing trial conduct, if any, and whether the process will be independent from investigators and the sponsor	26
**Ethics and dissemination**			
Research ethics approval	24	Plans for seeking research ethics committee/institutional review board (REC/IRB) approval	8, 28
Protocol amendments	25	Plans for communicating important protocol modifications (eg, changes to eligibility criteria, outcomes, analyses) to relevant parties (eg, investigators, REC/IRBs, trial participants, trial registries, journals, regulators)	26

For peer review only - http://bmjopen.bmj.com/site/about/guidelines.xhtml

Consent or assent	26a	Who will obtain informed consent or assent from potential trial participants or authorised surrogates, and how (see Item 32)	21
	26b	Additional consent provisions for collection and use of participant data and biological specimens in ancillary studies, if applicable	16, 21
Confidentiality	27	How personal information about potential and enrolled participants will be collected, shared, and maintained in order to protect confidentiality before, during, and after the trial	27
Declaration of interests	28	Financial and other competing interests for principal investigators for the overall trial and each study site	29
Access to data	29	Statement of who will have access to the final trial dataset, and disclosure of contractual agreements that limit such access for investigators	27
Ancillary and post-trial care	30	Provisions, if any, for ancillary and post-trial care, and for compensation to those who suffer harm from trial participation	Not applicable
Dissemination policy	31a	Plans for investigators and sponsor to communicate trial results to participants, healthcare professionals, the public, and other relevant groups (eg, via publication, reporting in results databases, or other data sharing arrangements), including any publication restrictions	8, 27
	31b	Authorship eligibility guidelines and any intended use of professional writers	27
	31c	Plans, if any, for granting public access to the full protocol, participant-level dataset, and statistical code	27
**Appendices**			
Informed consent materials	32	Model consent form and other related documentation given to participants and authorised surrogates	Separate
Biological specimens	33	Plans for collection, laboratory evaluation, and storage of biological specimens for genetic or molecular analysis in the current trial and for future use in ancillary studies, if applicable	15-17

*It is strongly recommended that this checklist be read in conjunction with the SPIRIT 2013 Explanation & Elaboration for important clarification on the items. Amendments to the protocol should be tracked and dated. The SPIRIT checklist is copyrighted by the SPIRIT Group under the Creative Commons "Attribution-NonCommercial-NoDerivs 3.0 Unported" license.

For peer review only - http://bmjopen.bmj.com/site/about/guidelines.xhtml

Pragmatic multicentre randomised controlled trial evaluating the impact of a routine molecular Point-of-Care 'test-and-treat' strategy for influenza in adults hospitalised with acute respiratory illness (FluPOC): trial protocol

Journal:	*BMJ Open*
Manuscript ID	bmjopen-2019-031674.R1
Article Type:	Protocol
Date Submitted by the Author:	20-Sep-2019
Complete List of Authors:	Beard, Kate; University of Southampton, Academic Unit of Clinical and Experimental Sciences, Faculty of Medicine<br>Brendish, Nathan ; University of Southampton, Academic Unit of Clinical and Experimental Sciences, Faculty of Medicine; University Hospital Southampton NHS Foundation Trust, Department of Infection<br>Malachira, Ahalya; University Hospital Southampton NHS Foundation Trust, Department of Infection<br>Mills, Samuel; University Hospital Southampton NHS Foundation Trust, Department of Infection<br>Chan, Cathleen; University Hospital Southampton NHS Foundation Trust, Department of Infection<br>Poole, Stephen; University of Southampton, Academic Unit of Clinical and Experimental Sciences, Faculty of Medicine; NIHR Southampton Biomedical Research Centre, University Hospital Southampton NHS Foundation Trust<br>Clark, Tristan; University of Southampton, Academic Unit of Clinical and Experimental Sciences, Faculty of Medicine; University Hospital Southampton NHS Foundation Trust, Department of Infection
**Primary Subject Heading**:	Infectious diseases
Secondary Subject Heading:	Respiratory medicine
Keywords:	Influenza, Adult, Hospitalised, Point-of-care test, Neuraminidase inhibitors

SCHOLARONE™ Manuscripts

Pragmatic multicentre randomised controlled trial evaluating the impact of a routine molecular Point-of-Care 'test-and-treat' strategy for influenza in adults hospitalised with acute respiratory illness (FluPOC): trial protocol

Kate R Beard*[1], Nathan J Brendish[1,2], Ahalya K Malachira[2], Samuel R Mills[2], Cathleen MT Chan[2], Stephen Poole[3], Tristan W Clark[1,2,3,4].

1. Academic Unit of Clinical and Experimental Sciences, Faculty of Medicine, University of Southampton, Southampton, UK

2. Department of Infection, University Hospital Southampton NHS Foundation Trust, Southampton, UK

3. NIHR Southampton Biomedical Research Centre, University Hospital Southampton NHS Foundation Trust, Southampton, UK

4. NIHR Post-doctoral Fellowship Programme, UK

Kate R Beard *Corresponding Author. LF100, Level F South Academic Block, Southampton General Hospital, Tremona Road, Southampton. SO16 6YD. Tel: 02381204888. Email: kate.beard@uhs.nhs.uk

Ahalya K Malachira: ahalya.malachira@uhs.nhs.uk, Nathan J Brendish: nathan.brendish@uhs.nhs.uk, Samuel R Mills: samuel.mills@uhs.nhs.uk, Cathleen MT Chan: cathleen.chan@uhs.nhs.uk, Stephen Poole: stephen.poole@nhs.net, Tristan W Clark: t.w.clark@soton.ac.uk

Word count: 4959

For peer review only

Items required by the World Health Organization Trial Registration Data Set

Trial Registration: SRCTN17197293

Date of registration: 13/11/2017

Secondary identifying numbers: RHM MED 1438, 17/SC/0368

Financial support: NIHR Post-doctoral Fellowship Programme: PDF-2016-09-061

Sponsor: University Hospital Southampton NHS Foundation Trust, (R&Doffice@uhs.nhs.uk)

Contact for public queries: Dr Tristan Clark, Associate Professor in Infectious Diseases, Clinical and Experimental Sciences, Faculty of Medicine, University of Southampton, Southampton, UK.

Tel: 02381208410. t.w.clark@soton.ac.uk

Contact for scientific queries: Dr Tristan Clark, Associate Professor in Infectious Diseases, Clinical and Experimental Sciences, Faculty of Medicine, University of Southampton, Southampton, UK.

Tel: 02381208410. t.w.clark@soton.ac.uk

Public title: Study examining the potential benefits of routine rapid accurate testing for influenza in adults with acute respiratory illness

Scientific title: Pragmatic multicentre randomised controlled trial evaluating the impact of a routine molecular Point-of-Care 'test-and-treat' strategy for influenza in adults hospitalised with acute respiratory illness (FluPOC): trial protocol

Countries of recruitment: UK

Health condition: Influenza

Intervention: Routine point-of-care testing with a rapid molecular test platform for influenza.

For peer review only

Control: Routine clinical care

Inclusion criteria:

- Patient in an ED* or AMU, within University Hospital Southampton NHS Foundation Trust (UHS) or Hampshire Hospitals NHS Foundation Trust.

- Aged ≥18 years old

- Has acute respiratory illness**

- Duration of respiratory illness less than 10 days prior to hospitalisation

- Can be recruited to the study within 16 hours of presentation

*For patients in ED a decision will have already been made that the patient will be admitted.

**An episode of acute respiratory illness is defined as an acute pulmonary illness (including pneumonia, bronchitis and influenza-like illness) or an acute exacerbation of a chronic respiratory illness (including exacerbation of COPD, asthma or bronchiectasis). For the study, acute respiratory illness as a provisional, working, differential or confirmed diagnosis must be made by a treating clinician.

Exclusion criteria:

- Not fulfilling all the inclusion criteria

- A purely palliative approach being taken by the treating clinicians

- Previously included in this study and re-presenting within the last 30 days after hospital discharge

- Declines nasal / pharyngeal swabbing

- Consent declined or consultee consent declined

For peer review only

Study type: Multicentre, randomised controlled trial. Open label, parallel group with 1:1 allocation to intervention and control groups.

Randomisation: Internet based random sequence allocation using random permuted blocks (sealedenvelope.com)

Date of first enrolment: 12/12/2017

Date of last enrolment: 03/05/2019

Initial target sample size: 840

Actual number recruited: 613

Recruitment status: completed

Primary Outcome: The primary outcome measure is the difference in the proportion of influenza positive patients treated appropriately with neuraminidase inhibitors (NAI) during their hospital stay (within 5 days of admission).

Key secondary outcomes*

Proportion of cases of influenza identified

Proportion of cases of all respiratory viruses detected

Proportion of all NAI use occurring in influenza positive patients

Proportion of all NAI use occurring in influenza negative patients

Time from admission to NAI commencement, hours

Duration of NAI use in influenza positive patients, days and doses

Duration of NAI use in influenza negative patients, days and doses

For peer review only

Proportion of patients treated with antibiotics

Proportion of patients treated with single doses or brief courses (<48 hours) of antibiotics

Duration of antibiotic use, days

Proportion of patients isolated in a side room

Duration of isolation facility use

Proportion of influenza cases correctly isolated

Time from admission to isolation of influenza positive cases

Time from admission to de-isolation of influenza negative cases

Duration of hospitalisation, days

Time to clinical stability, days

Time on supplementary oxygen, days

Proportion of patients with ICU or HDU admission

Duration of ICU or HDU stay, days

Proportion re-presenting to hospital within 30 days

Proportion readmitted to hospital within 30 days

In hospital, 30 and 60 day mortality

Turn-around time for viral testing

*All secondary outcome measures relate to the period of hospitalisation (up to 30 days from recruitment/admission)

For peer review only

PROTOCOL VERSION 1.1 23rd November 2017

Roles and responsibilities

Chief and Principal Investigator: Dr Tristan W Clark, Associate Professor in Infectious Diseases

Clinical and Experimental Sciences, Faculty of Medicine, University of Southampton, Southampton, UK and NIHR Southampton Biomedical Research Centre, University Hospital Southampton NHS Foundation Trust, Southampton, UK

Co-investigator: Dr Kate Beard, Clinical Research Fellow

Academic Unit of Clinical and Experimental Sciences, Faculty of Medicine, University of Southampton, Southampton, UK

Co-investigator: Dr Nathan J Brendish, Clinical Research Fellow

Academic Unit of Clinical and Experimental Sciences, Faculty of Medicine, University of Southampton, Southampton, UK and Department of Infection, University Hospital Southampton NHS Foundation Trust, Southampton, UK.

Co-investigator: Dr Ahalya Malachira, Clinical Research Fellow

Department of Infection, University Hospital Southampton NHS Foundation Trust, Southampton, UK.

Co-investigator: Dr Samuel Mills, Clinical Research Fellow

Department of Infection, University Hospital Southampton NHS Foundation Trust, Southampton, UK.

Co-investigator: Dr Cathleen Chan, Clinical Research Fellow

Department of Infection, University Hospital Southampton NHS Foundation Trust, Southampton, UK.

Co-investigator: Dr Stephen Poole, Clinical Research Fellow

For peer review only

NIHR Southampton Biomedical Research Centre, University Hospital Southampton NHS Foundation Trust

For peer review only

Abstract

Background

Influenza infections often remain undiagnosed in patients admitted to hospital due to lack of routine testing. When tested for, the diagnosis and treatment of influenza is often delayed due to the slow turnaround times of centralised laboratory PCR testing. Newer molecular systems, such as the FilmArray Respiratory Panel have comparable accuracy to laboratory PCR testing, and can generate a result in under 1 hour, making them potentially deployable as point-of-care tests (POCTs). High quality evidence for the impact of routine POCT for influenza on clinical outcomes is however, currently lacking. This large pragmatic multicentre randomised controlled trial aims to address this evidence gap.

Methods and analysis

The FluPOC trial is a pragmatic, multi-centre, randomised controlled trial evaluating adults admitted to a large teaching hospital and a district general hospital with an acute respiratory illness, during influenza season and defined by Public Health England (PHE). Up to 840 patients will be recruited over up to three influenza seasons, and randomised (1:1) to receive either POCT using the FilmArray Respiratory Panel, or routine clinical care. Clinical and infection control teams will be informed of the results in real time and where influenza is detected clinical teams will be encouraged to offered neuraminidase inhibitor (NAI) treatment in accordance with national guidelines. Those allocated to standard clinical care will have a swab taken for later analysis to allow assessment of missed diagnoses. The outcomes assessment will be by retrospective case note analysis. The outcome measures include the proportion of influenza-positive patients detected and appropriately treated with NAIs, isolation facility use, antibiotic use, length of hospital stay, complications and mortality.

Ethics and dissemination

For peer review only - http://bmjopen.bmj.com/site/about/guidelines.xhtml

Prior to commencing the study, approval was obtained from the South Central Hampshire A Ethics Committee (reference 17/SC/0368, granted 7th September 2017). Results generated from this protocol will be published in peer reviewed scientific journals and at presented at national and international conferences.

Trial Registration number

ISRCTN17197293

Key words

Influenza, Adult; Hospitalised; Point-of-care test; Neuraminidase inhibitors

For peer review only

Summary

Strengths and limitations of this study

- This is a multicentre randomised controlled trial
- The pragmatic trial design more accurately reflects routine clinical care in a hospital setting
- This is a highly relevant clinical question currently given the variable use of influenza testing and neuraminidase inhibitors in clinical practice, the ongoing threat of further influenza pandemics, the recent development of novel influenza antivirals, and the undefined role of influenza point-of-care testing in acute respiratory illness during seasonal influenza.
- The inclusion, of some patients lacking capacity and older patients often with multiple co-morbidities, makes the trial population generalizable to a secondary care population.
- There is ongoing uncertainty however as to how the suggested 'test-and-treat' strategy could be best implemented in clinical practice

For peer review only

For peer review only - http://bmjopen.bmj.com/site/about/guidelines.xhtml

Background

Respiratory virus burden of disease

Respiratory tract infections are the second most common cause of mortality and morbidity worldwide, [1] with viruses the most frequently detected pathogens in adults hospitalised with acute respiratory illness. [2] Seasonal influenza epidemics lead to excess hospitalisations and death due to complications including pneumonia and exacerbation of underlying cardiopulmonary conditions, and occur mainly in the elderly and patients with co-morbidity. [3-5]

Influenza: widespread but under-diagnosed

Estimates of the burden of influenza virus infection in hospitalised adults have traditionally been based on the incidence of the influenza-like-illness syndrome (ILI, defined as fever of >38°C and new respiratory symptoms) which has poor sensitivity (around 50%) and specificity (0-63%), rather than on laboratory confirmed influenza. [6-9] Patients may also present as decompensated cardiovascular disease, collapse or diabetic emergencies. [10,11] A recent Canadian study estimated that only around 1 in 14 emergency department (ED) visits due to influenza virus infection were correctly attributed to influenza. [12]

Sampling for respiratory viruses is generally performed on upper respiratory tract samples however several recent studies suggest that in lower respiratory tract syndromes (such as pneumonia and exacerbation of COPD) testing of upper respiratory tract samples for viruses is insensitive compared to testing lower respiratory tract samples. [13, 14]

For these reasons it is likely the burden of influenza and other respiratory viruses amongst hospitalised adults and its economic impact have been vastly under-estimated.

Neuraminidase inhibitors

For peer review only

Neuraminidase inhibitors (NAIs) such as oseltamivir (Tamiflu) are recommended by Public Health England (PHE) and WHO guidelines for all hospitalised adults with suspected and proven influenza infection. [15] Although there have been no randomised controlled trial evaluating the efficacy of NAIs in this group, well controlled observational data suggests that treatment of influenza with NAIs reduces mortality in hospitalised adults, especially when commenced rapidly. [16, 17] Therefore it is important that hospitalised patients with influenza are all identified and treated as soon as possible after presentation**.**

Previous POCTs for influenza

Rapid diagnostic tests for influenza, based on antigen detection in upper respiratory tract samples, have been available for many years but have poor diagnostic accuracy in adults, where sensitivity is around 50%. [18, 19] The poor sensitivity of these antigen-based tests has limited their clinical utility and their use was not associated with clinical or health economic benefits in a large randomised controlled trial in hospitalised adults. [20] The current gold standard diagnostic test for respiratory viruses is laboratory performed polymerase chain reaction (PCR) which is highly sensitive and specific but has a typical turnaround time for results of 24-48 hours. [21] New rapid, molecular tests have recently been developed, including the BioFire FilmArray Respiratory Panel. These molecular platforms are broadly equivalent in accuracy to laboratory PCR, without the need for specialist laboratory support and expertise, can provide a result in under 1 hour and can potentially be used as a point-of-care test (POCT).

FilmArray Respiratory Panel

The FilmArray Respiratory Panel 2 (BioFire Diagnostics, Utah, USA) uses nested real-time PCR to detect around 20 respiratory viruses. The FilmArray requires only 2 minutes of “hands on” time and produces a test result in around 45 minutes. [22] The FilmArray Respiratory Panel 2 is both FDA-cleared and CE IVD marked. The viral pathogens detected by the FilmArray Respiratory Panel 2

For peer review only

include: Influenza A (H1 and H3), Influenza B, Adenovirus, Coronaviruses (HKU1, NL63, 229E, OC43) Human Metapneumovirus, Human Rhinovirus/Enterovirus, Parainfluenza (types 1-4) and Respiratory Syncytial Virus [20, 21]. The FilmArray respiratory panel is broadly equivalent in accuracy to laboratory PCR, and has been validated on nose and throat swabs, nasopharyngeal aspirates, lower respiratory tract samples, and on samples from immunocompromised patients. [23-30] These studies also show high reliability, ease of use and short turnaround times.

Clinical impact of point-of-care testing for respiratory viruses

Though viruses are the most commonly detectable pathogen in adults hospitalised with acute respiratory illness, antibiotic use is near-universal. [2] Antibiotic over-use for acute respiratory illness is partly driven by clinical uncertainty regarding the underlying aetiology; therefore early identification of viruses may prevent or shorten unnecessary antibiotic use. Previous small studies have suggested that rapid molecular viral testing might reduce antibiotic use and improve influenza antiviral use. [31, 32] We have recently shown in a large pragmatic randomised controlled trial (ResPOC) that routine syndromic molecular POCT for respiratory viruses (using the FilmArray respiratory panel) in adults presenting to hospital with acute respiratory illness was associated with a number of clinical benefits compared to routine clinical care including a reduction in unnecessary antibiotics use, and a reduced length of hospital stay. It also suggested improved influenza detection and antiviral use and better side room utilisation, although the numbers of influenza infected patients was small and the study was not powered to evaluate outcomes in this subgroup. [33] This follow-on study seeks to definitively evaluate the impact of routine molecular POCT for influenza on the detection of influenza infected patients, their clinical care and outcome. Accepting the unpredictability of influenza activity year on year, this study is designed to recruit larger numbers of influenza infected patients, and powered to evaluate the impact of POCT on national guideline recommended antiviral use. In addition the novel use of sampling and deferred viral testing in the control group allows a direct assessment of missed diagnosis of influenza in hospitalised adults.

For peer review only

Alignment with global research priorities

In addition to being focused on patient and health care organisation outcomes, this clinical research is strongly aligned with several global research priority initiatives including the World Health Organisation's Battle against Respiratory Viruses (BRaVe) initiative [34] and the UK national report into antibiotic resistance. [35] The BRaVe initiative aims to catalyse multidisciplinary research on strategies to prevent and treat medically important respiratory virus infections with the goal of timely integration of research advances into public health practice. Priority areas identified include improving diagnostic tests for viral respiratory illness and improving the clinical management of patients with acute respiratory viral illness, both addressed by this study. With the growing threat of antimicrobial resistance to global public heath there is increasing need for the research and development of interventions which can help avoid unnecessary antibiotics use. [36] The data that will be generated by this trial regarding the impact of influenza POCT on antibiotic prescribing is highly aligned with this global initiative.

Added value

In addition to evaluating the clinical benefit of routine molecular POCT for influenza we will also use this opportunity to answer other important research questions including the ideal specimens for respiratory virus testing (upper versus lower respiratory tract specimens) in patients with lower respiratory tract infection, the difference in viral load between these two specimen type, and whether changes in viral load over time (kinetics) in respiratory samples or the presence of viral RNA in the blood, are related to and predictive of clinical outcome.

For peer review only

Study aims and objectives

Aims

This study aims to prospectively evaluate the impact of a routine molecular Point-of-Care ‘test and treat’ strategy for influenza in adults hospitalised with acute respiratory illness during influenza season.

Objectives

1. To evaluate the impact of routine molecular POCT on the clinical management of influenza including: the detection rate of influenza, the adherence to national (PHE) influenza guidelines, the speed and appropriateness of neuraminidase inhibitor use and the speed and appropriateness of isolation facility use.

2. To evaluate the impact of routine molecular POCT on measures of clinical effectiveness in adults hospitalised with influenza including: time to clinical stability, time on supplementary oxygen, duration of hospitalisation, adverse events and mortality.

3. To explore the differences in viral detection between upper and lower respiratory tract samples and the changes in viral load that occur over time in NAI treated and untreated patients.

Study processes that patient-participant will undergo are summarised in Figure 1.

For peer review only

Methods

Trial design

This is a pragmatic, multicentre, parallel group, open label, randomised controlled study. Groups are allocated 1:1 to the intervention arm (POCT) and control arm (routine clinical care). This protocol adheres to the SPIRIT statement. [37] The study will take place across up to three influenza seasons (as defined by national PHE surveillance programmes).

Participants, interventions and outcomes

Study setting

This is a multi-centre study based within secondary care at University Hospital Southampton NHS Foundation Trust, Southampton, UK and Royal Hampshire County Hospital, Hampshire hospitals NHS Foundation Trust, Winchester, UK. Patients will be recruited from the Acute Medical Unit (AMU) and the Emergency Department (ED) at these hospital sites.

Eligibility criteria

<u>Inclusion criteria</u>

- Is a patient in an ED* or AMU, within University Hospital Southampton NHS Foundation Trust (UHS) or Hampshire Hospitals NHS Foundation Trust.

- Aged ≥18 years old

- Has acute respiratory illness**

- Duration of respiratory illness less than 10 days prior to hospitalisation

For peer review only - http://bmjopen.bmj.com/site/about/guidelines.xhtml

- Can be recruited to the study within 16 hours of presentation

*For patients in ED a decision will have already been made that the patient will be admitted.

**An episode of acute respiratory illness is defined as an acute pulmonary illness (including pneumonia, bronchitis and influenza-like illness) or an acute exacerbation of a chronic respiratory illness (including exacerbation of COPD, asthma or bronchiectasis). For the study, acute respiratory illness as a provisional, working, differential or confirmed diagnosis must be made by a treating clinician.

<u>Exclusion criteria</u>

- Not fulfilling all the inclusion criteria

- A purely palliative approach being taken by the treating clinicians

- Previously included in this study and re-presenting within the last 30 days after hospital discharge

- Declines nasal / pharyngeal swabbing

- Consent declined or consultee consent declined

Concurrent, prior or subsequent enrolment in an observational study is not necessarily an exclusion criterion; this is at the discretion of the chief investigator and will be assessed on a case-by-case basis.

Interventions

<u>For both control and intervention groups:</u>

A respiratory sample will be collected by a member of research staff. A combined nose and throat swab will be taken and placed into viral transport medium. If feasible, a lower respiratory tract sample (sputum) will also be obtained.

For peer review only

Clinical and demographic data will be collected at the time of enrolment. Outcome data will be collected from case notes and electric health records retrospectively.

A blood sample (maximum of 21ml) will be obtained from all willing participants, processed in the NIHR Wellcome Trust Clinical Research Facility lab and stored at -80°C pending further analysis. All respiratory samples left-over from FilmArray testing may be frozen and stored for further study. Participant consent for this is included in the consent form. Patients may be approached for additional samples to be collected and stored for further study (see below for schedule of additional samples).

A letter detailing that the patient has been included in this trial will be sent to the patient's general practitioner for information only.

<u>For those randomised to the interventional arm:</u>

The nose and throat swab in viral transport medium will be analysed promptly on the FilmArray Respiratory Panel 2 as per training delivered by the apparatus manufacturer, used as a point-of-care test. FilmArray machines will be located in the clinical areas for this purpose. Test results are generated in around 45 minutes. Where lower respiratory tract samples are also obtained these will also be tested on the FilmArray in parallel with the nose and throat swab or sequentially.

The results of the test will be documented in the patient's case notes and a member of the clinical team responsible for the patient will be directly informed. The participant or consultee will also be informed of the result where appropriate. The infection prevention and control team will be informed of the result within 4 hours of testing. In the event of influenza A or B being detected clinical teams will be encouraged to offered treatment with NAIs as per national guidelines. It is known that the sensitivity of virus detection may be greater with lower respiratory tract samples versus upper tract samples in certain patients. Therefore in patient where lower respiratory tract samples are obtained in addition to the upper respiratory tract samples, if either of the swabs are

For peer review only - http://bmjopen.bmj.com/site/about/guidelines.xhtml

positive they will treated as infected and be reported to the clinical team and infection control team as above.

For those randomised to the control group

These patients will be managed according to standard clinical care as directed by their treating clinicians. Laboratory respiratory virus testing will be at the discretion of the responsible clinical team and where performed will be using laboratory PCR (in the onsite laboratory). The respiratory samples collected at enrolment will be stored at -80°C and subsequently tested by the FilmArray respiratory panel at least 30 days after collection. This will allow direct comparison of pathogens between the groups (i.e. an estimate of missed diagnoses in the routine clinical care group) but will not influence participant care.

Additional procedures

For participants randomised to receive the intervention, who subsequently have a pathogen detected on the FilmArray respiratory panel and for all participants randomised to routine clinical care, additional respiratory samples +/- an additional blood test (maximum 10mls) may be specifically requested from the participants to study temporal changes in viral load, according to the schedule below.

Schedule

The nose and throat swab and lower respiratory tract samples listed here are in addition to samples taken at enrolment (day 1) and are collected at five further time points: day 2 to 3, day 4 to 5, day 6 to 7, day 8 to 10 and day 11-14+. An additional blood sample (maximum of 10mls) may be taken at day 5 to 7. The participant may decline all or any of these additional tests.

Primary outcome

For peer review only

The primary outcome measure is the difference in the proportion of influenza positive patients treated appropriately with neuraminidase inhibitors (NAI) during their hospital stay (within 5 days of admission).

Secondary Outcomes

Proportion of cases of influenza identified

Proportion of all NAI use occurring in influenza positive patients

Proportion of all NAI use occurring in influenza negative patients

Median time from admission to NAI commencement, hours

Median duration of NAI use in influenza positive patients, days and doses

Median duration of NAI use in influenza negative patients, days and doses

Proportion of patients treated with antibiotics

Proportion of patients treated with single doses or brief courses (<48 hours) of antibiotics

Median duration of antibiotic use, days

Proportion of patients isolated in a side room

Median duration of isolation facility use

Proportion of influenza cases correctly isolated

Median time from admission to isolation of influenza positive cases

Median time from admission to de-isolation of influenza negative cases

Median duration of hospitalisation, days

Median time to clinical stability*, days

Median time on supplementary oxygen, days

Proportion of patients with ICU or HDU admission

Median duration of ICU or HDU stay, days

Proportion re-presenting to hospital within 30 days

Proportion readmitted to hospital within 30 days

Proportion of in-hospital, 30 and 60 day mortality

For peer review only

Median turn-around time for viral testing

All outcomes are measured for the duration of hospitalisation or up to 30 days from recruitment/admission (whichever is shortest) unless specified otherwise and include medication (antibiotics and NAIs) that patients are discharged home with.

*Defined as: temperature ≤37.8C, respiratory rate ≤24 breaths per minute, heart rate ≤100 beats per minute, oxygen saturation ≥90% without the use of supplementary oxygen, systolic blood pressure ≥90 and normal mental status mmHg for at least 24 hours. [38]

Exploratory Outcomes

Difference in influenza detection between upper and lower respiratory tract samples

Difference in influenza viral load between upper and lower respiratory tract samples

Proportion with influenza RNA detected in blood

Changes in influenza viral load over time (kinetics) in respiratory tract samples and blood

Response rate on the ordinal hospital recovery scale (HRS) at day 5 and/or 7

Participant Timeline

Participants are identified in the AMU and ED according to eligibility criteria by research staff. Following gaining written consent participants are immediately randomised to the intervention group or the control group. For those patients randomised to the intervention group a nose/throat swab is collected by a study doctor or research nurse and this is tested on the FilmArray machine. If the patient is able to produce a sputum sample, this sample will also be tested on the FilmArray machine. Results are generated in approximately 45 minutes and are immediately communicated to the clinical team. Clinical data is collected prospectively and retrospectively for both groups. Further nose/throat swabs, sputum samples and blood tests may be collected from patients who remain inpatients on serial follow up visits up to day 14.

For peer review only - http://bmjopen.bmj.com/site/about/guidelines.xhtml

Sample size

Proposed sample size

The study will recruit up to 840 patient-participants: about 280 per season for three consecutive influenza seasons. With 1:1 allocation to groups, there will be 420 patients per group. On the conservative assumption of a 25% positivity rate for influenza during influenza season (i.e. ~100 influenza positive patients in each group) the group sizes would give a 90% power at a 0.05 significance level to detect a difference of 20% in appropriate neuraminidase inhibitor use (from 65% to 85%) and an 80% power to detect a difference of 17%. In our previous work 91% of influenza positive patients randomised to POCT were appropriately treated with NAI versus 65% with routine clinical care, a difference of 26% (95%CI 10 to 43). We suspect that the difference may be larger in this study as there will likely be a number of undiagnosed (and therefore untreated) cases in the control group revealed by testing them with the FilmArray at a later date. In the event of higher numbers of influenza positive patients being recruited in the first two seasons, the study may be stopped prematurely (once ~100 influenza positive patients have been recruited to the intervention group).

Recruitment and Screening

Screening

Eligible patients in the acute medical unit (AMU) and the emergency department will be identified by research staff via regular review of the comprehensive admissions IT systems daily. Recruitment will run for up to 3 years to include 3 seasonal influenza seasons between December 2017 and May 2020.

For peer review only - http://bmjopen.bmj.com/site/about/guidelines.xhtml

Consent

The study team (research nurse, research fellow or the principal investigator) will obtain informed consent for those fulfilling eligibility criteria and willing to be recruited for those with capacity, or assent via consultee for those without capacity, as per the dedicated study forms. In view of the acute nature of patients' illnesses, the potential benefits of rapid identification of viruses including prompt neuraminidase inhibitor use for influenza, the usual 24 hour consideration period for a participant or consultee will not apply.

Discussion of the study will be provided to patients, or their consultee for those lacking capacity, by study staff. This includes supply of a participant information sheet for the participant or witness to read and retain. Consent is also acquired to obtain and store specimens (nose/throat swab, sputum sample, blood tests) for future research studies.

Each patient will be assumed to have capacity unless it is established that they lack capacity. For patients unable to consent for themselves, this study complies with the Mental Capacity Act 2005 and in such cases, the patient's family member, carer or friend may be asked to act as the personal consultee and provide assent. In the event of a personal consultee not being available a nominated consultee (usually the consultant caring for the patient and independent from the study) will be asked if they would provide assent. Both the person taking assent and the consultee must personally sign and date the relevant form.

For peer review only

Assignment of interventions

Sequence generation, allocation concealment and implementation

Following screening and obtaining informed written consent from an eligible patient, a unique identification number will be assigned consecutively. A research team member will then use this identification number to randomise the participant to either the intervention or control group, via an internet-based randomisation service (sealedenvelope.com). This service uses permuted blocks of

varying sizes and generates a randomisation code which corresponds to one of the study arms. Research staff will use the allocation code generated from sealedenvelope.com to assign the patients to the intervention or control group.

Blinding

As this is a pragmatic trial of a diagnostic strategy where the results of the test must be communicated to clinical and infection control teams no attempt at blinding trial participants, research staff or care providers will be made. Data analysts will be blinded to group allocation.

For peer review only

Data collection, management and analysis

Data collection methods

Demographic and clinical data will be collected for all patients at enrolment from paper and electronic medical records including: age, sex, ethnicity, smoking status, vaccination status, co-morbidities, medication use, symptoms, duration of illness prior to hospitalisation, observations (pulse rate, respiratory rate, blood pressure, oxygenation status), laboratory results, radiology results, antimicrobial and antiviral use prior to hospitalisation and provisional diagnosis. Data will be recorded on a standardised Case Report Form (CRF) and transferred to a secure electronic database. Once patients have been discharged or after 30 days (whichever is soonest), outcome data will be collected retrospectively from electronic and physical case notes, electronic prescribing systems and laboratory and radiological results systems including: use of antivirals, duration of antivirals, time from assessment to antiviral use, use of antibiotics, duration of antibiotic use, use of side room facilities, time from assessment to isolation facility use, time to clinical stability, duration of supplementary oxygen use, duration of hospitalisation, complications including ICU and HDU admission, representation and readmission to hospital within 30 days, final diagnosis and mortality. The number of diagnostic tests and procedures performed will also be recorded and data will also be

For peer review only - http://bmjopen.bmj.com/site/about/guidelines.xhtml

collected on the turnaround time of respiratory virus test results in each group. Patients withdrawn from the study will have no further data collected.

Data Management

The study will be conducted in compliance with the approved protocol, relevant International Conference on Harmonisation (ICH) and Good Clinical Practice (GCP) regulations and standard operating procedures. Data will be evaluated for protocol compliance and accuracy in reference to the source documents.

The subjects' anonymity will be maintained. The study team will keep a log of each subject's name, hospital ID number, date of birth, and unique participant trial number. The participant details will be recorded on the secure NHS Edge system in a similar manner, including NHS number. This participant trial number is used on documents after screening to maintain confidentiality. Documents that are not anonymous (e.g. signed informed consent forms) will be maintained separately, in strict confidence and in accordance with the Data Protection Act 1998. A secure database will be used to enter data from the CRFs at the completion of the study, followed by data lock.

The study staff will be responsible for entering study data in the CRF. It is the investigators' responsibility to ensure the accuracy of the data entered in the CRF.

Only the research study team will know the identity of subjects and have access to the list linking participant details to the participant trial number.

Statistical methods

For peer review only

Analysis will be by intention-to-treat. The framework is superiority. Trial results will be reported in accordance with the CONSORT statement. [39]

Statistical analysis will be performed by a dedicated medical statistician from the University of Southampton, independent from the study team. No interim analysis is currently planned. Patients tested with the rapid molecular diagnostic test will be compared with patients managed according to standard clinical care using standard descriptive and comparative statistical methods using Prism (GraphPad Software Inc; La Jolla, California), and Stata (StataCorp, College Station, Texas).

Missing data was minimal (<2%) in the CI's previous POCT study involving 720 patients presenting to secondary care, and is not expected to be significant higher in this trial, however the use of multiple imputation will be performed should missing data exceed 5% for the primary outcome or key secondary outcome measures. [40]

Summaries of all baseline characteristics will be presented using means and standard deviations, medians and interquartile ranges, or frequencies and percentages, as appropriate. The intervention and control groups will be compared using odds ratios and differences in proportions for equality of proportions for binary data (e.g. proportion of cases of influenza detected) and using independent-samples t-tests or non-parametric equivalent as appropriate for continuous data (e.g. duration of antibiotics, test turn-around time etc).

For peer review only

Primary outcome

The primary outcome (proportion of influenza infected patients treated with NAIs) will be measured only in patients with confirmed influenza, ie the intention-to-treat infected (ITTI) population. Difference in proportions and unadjusted odds ratios will be used to compare the groups. The effect of group on the primary outcome will be further assessed using multiple logistic regression to

control for the following covariates: age, sex, influenza vaccine status, receipt of antibiotics prior to admission, duration of illness, co-morbidity, temperature, CRP, severity of illness, and clinical group.

Certain secondary outcome measures will be measured both in the entire cohort (ITT population) and in the influenza infected cohort (the ITTI population) whilst others will only be measured in the ITTI population, as detailed in the outcome section. The intervention and control groups will be compared as per the primary outcome for equality of proportions for binary data and using t-tests and non-parametric equivalent tests for continuous data (e.g. turnaround time) as appropriate.

In view of the clinical heterogeneity of influenza infected patients, heterogeneity of effect among different clinical subgroups is anticipated *a priori* and therefore a pre-planned subgroup analysis for key secondary outcome measures will be undertaken based on clinical group (exacerbation of asthma, exacerbation of COPD, pneumonia, ILI, non-pneumonic LRTI and 'other') and also based on receipt or otherwise of antiviral treatment (the receipt of NAIs within 5 days of admission). The interaction between clinical subgroups and allocation group will be assessed in the multiple regression models detailed above.

Monitoring

This trial was reviewed by the sponsor. On the basis of the very low risk of harms associated with the intervention in this non-CTIMP trial, no data monitoring committee or interim analysis is planned. A trial management committee will meet regularly and oversee the trial.

Harms

For peer review only

The risks of respiratory tract sampling and additional blood tests being taken are minimal and where occurring are likely to be mild. No additional adverse events related to POCT for respiratory viruses are anticipated. However active monitoring and reporting of severe adverse events will be undertaken. A Serious Adverse Event (SAE) is any adverse event that:

- Results in death
- Is life-threatening
- Requires hospitalisation or prolongation of existing hospitalisation
- Results in persistent or significant disability or incapacity
- Consists of a congenital anomaly or birth defect

Participants already admitted to AMU or are in ED but with a decision already made to admit, are considered already hospitalised. However, an adverse event leading to prolongation of their existing hospitalisation will be counted as an SAE.

Auditing

Regular monitoring by the sponsor will be performed according to ICH GCP. Data will be evaluated with regards to accuracy in relation to source documents and compliance with the protocol. Following written standard operating procedures, the monitors will verify that the clinical trial is conducted and data are generated, documented and reported in a manner that is compliant with the protocol, GCP and applicable regulatory requirements.

Protocol amendments

For peer review only

There has been one amendment made to the protocol. This was the addition of Hampshire Hospitals NHS Foundation Trust as a potential site and removal of mention of 'single centre.' The current protocol version is 1.1, dated 23rd November 2017. This amendment has been communicated to the investigators and the trial registries. The local study reference is RHM MED 1438.

Confidentiality

All data will be anonymised to ensure patient confidentiality is protected. A unique study number will be used to identify participant data in the CRFs and database. Serious Adverse Events will be reported in line with GCP and regulatory requirements. All study staff have been trained in GCP. Data will be kept securely and only the investigators and sponsor's representative (monitor) have access to the data.

Access to data

Full access to the data set will be held by the principal investigator, co-investigators and independent statistician only. The data set may be made available to other parties on request.

Dissemination policy

Authorship of this manuscript and those resulting from this protocol will follow the ICMJE recommendations and CONSORT statement where appropriate, and there are no plans to use professional writers. There is no intention to make the data set publically available. Outside of the study team and regulators, the full protocol will only be made available at the discretion of the chief

For peer review only

investigator. Multiple publications are expected to result from the data and samples collected, and these publications must acknowledge this trial and the study team as appropriate.

Patient and public involvement

Discussion with members of the public and the results of questionnaire from hospitalised patients helped inform the design of this trial.

The emphasis on informing the patients, in addition to the clinical teams, of the POCT result is a direct result of our engagement with patients. Patients and the public will also be involved in the reporting of this research.

Discussion

We hypothesise that the use of a routine molecular POC 'test and treat' strategy for influenza in adults hospitalised with acute respiratory illness will improve adherence to national guidelines for the management of influenza and may improve patient outcomes. We expect increased, timely detection of influenza and that this will lead to improved use of NAI treatment. Rapid and appropriate use of NAI treatment in influenza may also lead to decreased complication and mortality and may shorten length of stay in hospital. The rapid detection of viruses may improve isolation facility use within the NHS with subsequent reduction in nosocomial transmission of viruses and improvements in patient flow through acute areas. The use of a POCT for respiratory viruses may also identify patients where unnecessary antibiotic use can be safely discontinued, promoting antibiotic stewardship and reducing antimicrobial resistance.

Declarations

Ethics approval

For peer review only

Prior to commencing the study, approval was obtained from the South Central Hampshire A Ethics Committee (reference 17/SC/0368). A protocol amendment has been approved by the ethics committee for the addition of Hampshire Hospitals NHS Foundation Trust as a potential site and the removal of mention of 'single centre' (current protocol version 1.1, date 23rd November 2017). The local study reference number is RHM MED 1438.

Consent for publication

Not applicable

Availability of data and material

Not applicable

Competing interests

The principal investigator and co-investigators declare they have no competing interests relating to this study.

Funding

The study is funded by NIHR Post-doctoral Fellowship Programme, grant number: PDF-2016-09-061. The testing units and equipment for this study will be leased or purchased independently from a UK distributor using funds provided by NIHR Post Doctoral Fellowship Programme (PDF-2016-09-061). NIHR Clinical Research Network, Wessex provided salary contributions for clinical fellows to support this trial. The study is sponsored by University Hospital Southampton NHS Foundation Trust.

The study sponsor, study funders and manufacturers of the FilmArray platform had no involvement in the conception, design or running of this study, and will have no involvement in the analysis of the data or writing of subsequent manuscripts for publication.

For peer review only

The views expressed in this publication are those of the author(s) and not necessarily those of the NHS, the National Institute for Health Research or the Department of Health.

Author's Contributions

TWC conceived the study and is the chief and principal investigator. TWC and NJB designed the study. TWC and NJM wrote the protocol. KRB wrote the protocol manuscript. AKM, SRM, CC and SP reviewed the manuscript. All authors read and approved the final manuscript.

Acknowledgments

We would like to thank patients and staff in the Emergency Department and Acute Medical Unit and other inpatient wards at University Hospital Southampton NHS Foundation Trust and Hampshire Hospitals NHS Foundation Trust for making the study possible.

Abbreviations

AE: Adverse Event

AMU: Acute medical unit

CES: Clinical and Experimental Sciences academic unit, University of Southampton

CI: Chief Investigator

CRF: Case Report Form

ED: Emergency department

ICU: Intensive care unit

For peer review only

HDU: High dependency unit

HRA: Health Research Agency

NAI: Neuraminidase inhibitor

(NIHR) WTCRF: NIHR Wellcome Trust Clinical Research Facility

PHE: Public Health England

PI: Principal Investigator

POCT: Point-of-Care Test

REC: Regional Ethics Committee

SAE: Serious Adverse Event

UHS: University Hospital Southampton NHS Foundation Trust

References

1. Lozano R, Naghavi M, Foreman K, et al. Global and regional mortality from 235 causes of death for 20 age groups in 1990 and 2010: a systematic analysis for the Global Burden of Disease Study 2010. *Lancet* 2012;380:2095–128.

2. Clark TW, Medina MJ, Batham S, et al. Adults hospitalised with acute respiratory illness rarely have detectable bacteria in the absence of COPD or pneumonia; viral infection predominates in a large prospective UK sample. *J. Infect* 2014;69:507–15.

3. Thompson WW, Shay DK, Weintraub E, et al. Mortality associated with influenza and respiratory syncytial virus in the United States. *JAMA* 2003;289:179–86.

For peer review only

4. Thompson WW, Shay DK, Weintraub E, et al. Influenza-associated hospitalizations in the United States. *JAMA* 2004;292:1333–40.

5 Irwin DE, Weatherby LB, Huang WY, et al. Impact of patient characteristics on the risk of influenza/ILI-related complications. *BMC Health Serv Res* 2001;1:8.

6. Dool C van den, Hak E, Wallinga J, et al. Symptoms of influenza virus infection in hospitalized patients. *Infect Control Hosp Epidemiol* 2008;29:314–9.

7. Babcock HM, Merz LR, Dubberke ER, et al. Case-control study of clinical features of influenza in hospitalized patients. *Infect Control Hosp Epidemiol* 2008;29:921–6.

8. Babcock HM, Merz LR, Fraser VJ. Is influenza an influenza-like illness? Clinical presentation of influenza in hospitalized patients. *Infect Control Hosp Epidemiol* 2006;27:266–70.

9. Call SA, Vollenweider MA, Hornung CA, et al. Does this patient have influenza? *JAMA* 2005;293:987–97.

10. Falsey AR, Hennessey PA, Formica MA, et al. Respiratory syncytial virus infection in elderly and high-risk adults. *N Engl J Med* 2005;352:1749–59.

11. Warren-Gash C, Smeeth L, Hayward AC. Influenza as a trigger for acute myocardial infarction or death from cardiovascular disease: a systematic review. *Lancet Infect Dis* 2009;9:601–10.

12. Schanzer DL, Schwartz B. Impact of seasonal and pandemic influenza on emergency department visits, 2003-2010, Ontario, Canada. *Acad Emerg Med* 2013;20:388–97.

13. López Roa P, Rodríguez-Sánchez B, Catalán P, et al. Diagnosis of influenza in intensive care units: lower respiratory tract samples are better than nose-throat swabs. *Am J Respir Crit Care Med* 2012;186:929-30.

For peer review only

14. Falsey AR, Formica MA, Walsh EE. Yield of sputum for viral detection by reverse transcriptase PCR in adults hospitalized with respiratory illness. *J Clin Microbiol* 2012;50:21-4.

15. PHE guidance on use of antiviral agents for the treatment and prophylaxis of seasonal influenza. Public Health England (2015-16). Version 6.0. September 2015.

16. Muthuri SG, Venkatesan S, Myles PR, et al. Effectiveness of neuraminidase inhibitors in reducing mortality in patients admitted to hospital with influenza A H1N1pdm09 virus infection: a meta-analysis of individual participant data. *Lancet Respir Med* 2014;2:395–404.

17. Katzen j, Kohn R, Houk JL et al. Early Oseltamivir After Hospital Admission Is Associated With Shortened Hospitalization: A 5-Year Analysis of Oseltamivir Timing and Clinical Outcomes. *Clin Infect Dis* 2019;69(1):52-58.

18. Evaluation of rapid influenza diagnostic tests for detection of novel influenza A (H1N1) Virus - United States, 2009. *MMWR Morb Mortal Wkly Rep* 2009;58:826–9.

19. Chartrand C, Leeflang MM, Minion J, et al. Accuracy of rapid influenza diagnostic tests: a meta-analysis. *Ann Intern Med* 2012;156:500–11.

20. Nicholson KG, Abrams KR, Batham S, et al. Randomised controlled trial and health economic evaluation of the impact of diagnostic testing for influenza, respiratory syncytial virus and Streptococcus pneumoniae infection on the management of acute admissions in the elderly and high-risk 18- to 64-year-olds. *Health Technol Assess* 2014;18:1e274.

21. Mackay IM. Real-time PCR in the microbiology laboratory. *Clin Microbiol Infect* 2004;10:190–212.

22. BioFire Diagnostics. FilmArray® Respiratory Panel Information Sheet.

23. Popowitch EB, O'Neill SS, Miller MB. Comparison of the Biofire FilmArray RP, Genmark eSensor RVP, Luminex xTAG RVPv1, and Luminex xTAG RVP fast multiplex assays for detection of respiratory viruses. *J Clin Microbiol* 2013;51:1528–33.

For peer review only - http://bmjopen.bmj.com/site/about/guidelines.xhtml

24. Poritz MA, Blaschke AJ, Byington CL, et al. FilmArray, an automated nested multiplex PCR system for multi-pathogen detection: development and application to respiratory tract infection. *PLoS ONE* 2011;6:e26047.

25. Butt SA, Maceira VP, McCallen ME, et al. Comparison of three commercial RT-PCR systems for the detection of respiratory viruses. *J Clin Virol* 2014;61:406–10.

26. Pierce VM, Elkan M, Leet M, et al. Comparison of the Idaho Technology FilmArray system to real-time PCR for detection of respiratory pathogens in children. *J Clin Microbiol* 2012;50:364–71.

27. Loeffelholz MJ, Pong DL, Pyles RB, et al. Comparison of the FilmArray Respiratory Panel and Prodesse Real-Time PCR Assays for Detection of Respiratory Pathogens. *J Clin Microbiol* 2011;49:4083-8.

28. Ruggiero P, McMillen T, Tang YW, et al. Evaluation of the BioFire FilmArray Respiratory Panel and the GenMark eSensor Respiratory Viral Panel on Lower Respiratory Tract Specimens. *J Clin Microbiol* 2014;52:288-90.

29. Hammond SP, Gagne LS, Stock SR, et al. Respiratory virus detection in immunocompromised patients with FilmArray respiratory panel compared to conventional methods. *J Clin Microbiol* 2012;50:3216–21.

30. Doern CD, Lacey D, Huang R, et al. Evaluation and Implementation of FilmArray Version 1.7 for Improved Detection of Adenovirus Respiratory Tract Infection. *J Clin Microbiol* 2013;51:4036-9.

31. Branche AR, Walsh EE, Vargas R, et al. Serum Procalcitonin Measurement and Viral Testing to Guide Antibiotic Use for Respiratory Infections in Hospitalized Adults: A Randomized Controlled Trial. *J Infect Dis* 2015;212:1692-700.

32. Dugas AF, Valsamakis A, Atreya MR, et al. Clinical diagnosis of influenza in the ED. *Am J Emerg Med* 2015;33:770-5.

For peer review only

33. Brendish NJ, Malachira AK, Armstrong L, et al. Routine molecular point-of-care testing for respiratory viruses in adults presenting to hospital with acute respiratory illness (ResPOC): a pragmatic, open-label, randomised controlled trial. *Lancet Respir Med* 2017;5:401-411.

34. Legand A, Hayden FG. Addressing the public health burden of respiratory viruses: the Battle against Respiratory Viruses (BRaVe) Initiative. *Future Virology* 2013;8:953–968.

35. Jim O'Neil (chair). Tackling drug-resistant infections globally: Final report and recommendations. 2016.

36. WHO. Fact sheet: antimicrobial resistance.https://www.who.int/en/news-room/fact-sheets/detail/antimicrobial-resistance (accessed 4th May 2019)

37. Chan A-W, Tetzlaff JM, Altman DG, et al. SPIRIT 2013 Statement: Defining standard protocol items for clinical trials. *Ann Intern Med* 2013;158:200-207.

38. Halm EA, Fine MJ, Marrie TJ, et al. Time to clinical stability in patients hospitalized with community-acquired pneumonia: implications for practice guidelines. *JAMA* 1998;279:1452–1457.

39. Schulz KF, Altman DG, Moher D, for the CONSORT Group. CONSORT 2010 Statement: updated guidelines for reporting parallel group randomised trials. *BMC Med* 2010;8:18.

40. Jakobsen JC, Gluud C, Wetterslev J, Winkel P. When and how should multiple imputation be used for handling missing data in randomised clinical trials – a practical guide with flowcharts. *BMC Med Res Methodol* 2017 Dec 6;17(1):162

Figure 1 Participant flow through the study

For peer review only

For peer review only

For peer review only - http://bmjopen.bmj.com/site/about/guidelines.xhtml

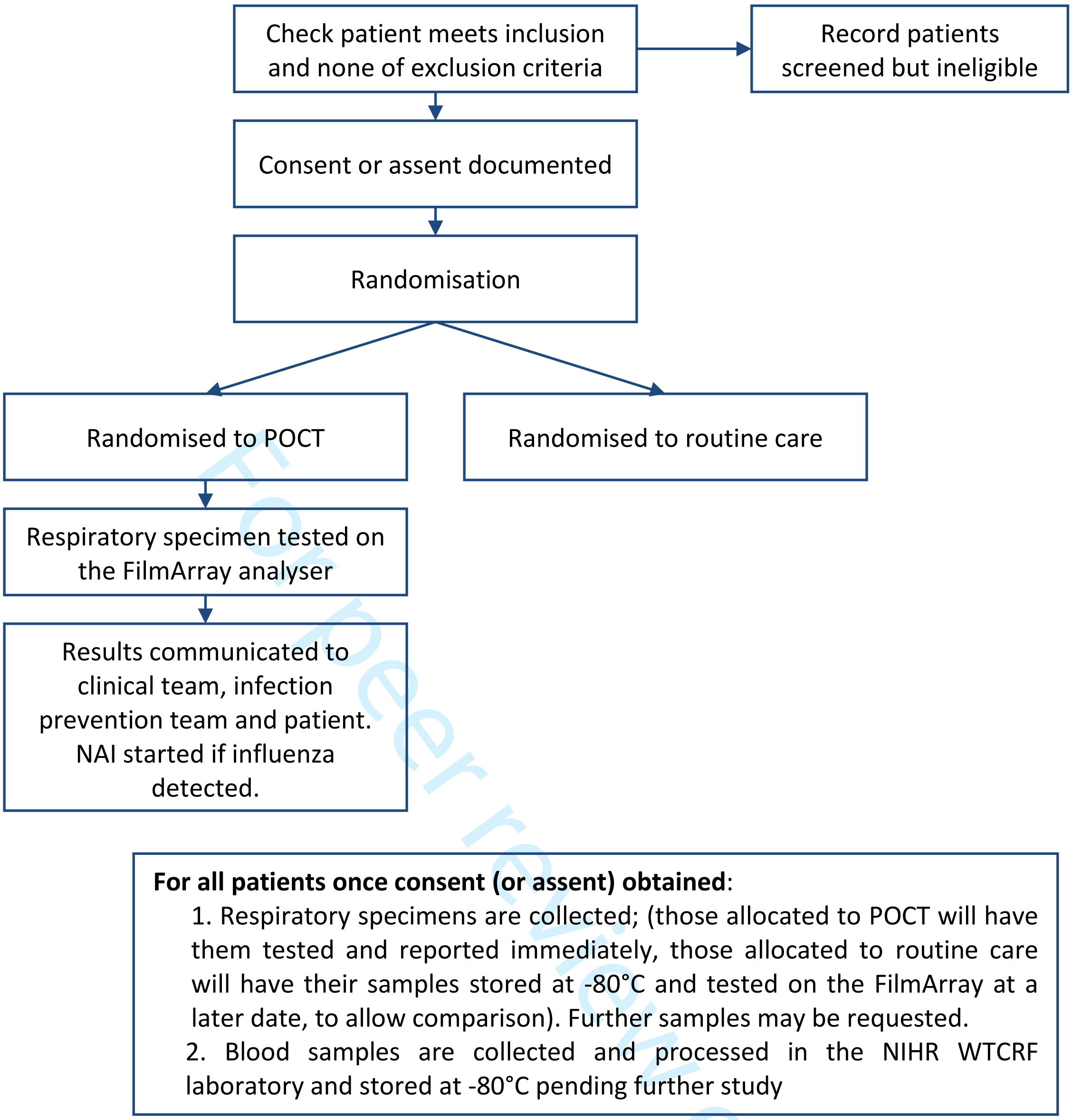

For peer review only
